# An Embedded Multi-Sensor IoT Platform for Agricultural Monitoring Using Meshtastic-Based LoRa Communication and Cloud Microservices

**DOI:** 10.3390/s26185839

**Published:** 2026-09-15

**Authors:** Cătălin Negulescu, Theodor Borangiu, Silviu Răileanu, Victor-Valentin Anghel

**Affiliations:** 1 National University of Science and Technology POLITEHNICA Bucharest, 060042 Bucharest, Romania; theodor.borangiu@upb.ro (T.B.); silviu.raileanu@upb.ro (S.R.); victor.anghel@stud.acs.upb.ro (V.-V.A.); 2 Academy of Romanian Scientists, 050044 Bucharest, Romania

**Keywords:** smart agriculture, LoRa mesh, IoT technologies, cloud computing, microservices

## Abstract

The paper presents a low-cost agricultural monitoring platform based on embedded IoT sensing, LoRa mesh communication, and an Edge–Fog–Cloud architecture designed for real-time environmental monitoring in smart agriculture applications. The proposed system integrates distributed sensor nodes built around the Heltec Mesh Node T114 platform, combining an nRF52840 microcontroller with an SX1262 LoRa transceiver to support low-power, long-range communication in agricultural environments. Environmental data acquisition is performed using integrated BME280 temperature, humidity, and pressure sensors, capacitive soil moisture sensors, and TEMT6000 light sensors. A custom firmware architecture extending the Meshtastic framework was developed to support sensor integration, telemetry generation, packet forwarding, and energy-aware scheduling within the LoRa mesh network. The platform combines embedded telemetry modules, intermediate routing mechanisms, and cloud-based microservices responsible for data aggregation and processing. Experimental results showed stable radio-link conditions in the tested node-to-router configuration, with RSSI values around −55 dBm and SNR values of approximately 6–7 dB. Channel utilization varied between approximately 4% and 12%, while transmission airtime utilization remained below 5%. These results support the feasibility of the proposed sensing, communication, and data-processing architecture under the tested configuration. The architecture is designed to accommodate additional sensing and routing nodes, while multi-hop performance, long-term energy consumption, and network scalability remain to be evaluated in larger deployments.

## 1. Introduction

Digital transformation has become an essential component of modern agriculture, enabling the transition from conventional farming practices toward data-driven and precision-oriented agricultural systems. In recent years, the integration of Internet of Things (IoT) technologies, embedded sensing platforms, cloud computing, and artificial intelligence has significantly improved the capability of monitoring environmental conditions, optimizing resource utilization, and supporting real-time decision-making processes in agricultural environments [1,2,3,4]. Continuous monitoring of parameters such as temperature, humidity, soil moisture, atmospheric pressure and light intensity allows farmers to better understand field conditions and apply targeted interventions aimed at improving productivity while reducing operational costs and environmental impact. Precision agriculture technologies further contribute to reducing water consumption, improving fertilization strategies, and increasing sustainability through real-time environmental telemetry and automated monitoring systems [1,2,4].

In practice, the deployment of such technologies remains challenging, particularly for small- and medium-sized farms. Agricultural monitoring systems often operate over large areas with limited power infrastructure and intermittent connectivity, while proprietary or cloud-dependent solutions may increase deployment cost and reduce flexibility. These constraints motivate the use of low-power, long-range communication technologies such as LoRa and LoRaWAN for remote environmental telemetry [5,6].

Recent agricultural IoT systems increasingly combine low-power sensing, long-range communication, and distributed Edge–Fog–Cloud processing; the most relevant approaches are reviewed in Section 2 [7,8,9,10,11,12,13].

However, many existing implementations remain strongly focused on cloud-level analytics while providing limited details regarding embedded firmware architecture, telemetry generation mechanisms, packet forwarding strategies, energy-aware scheduling, and the practical integration of low-cost sensing hardware within real LoRa deployments. In addition, most available systems remain dependent on centralized LoRaWAN infrastructures or proprietary cloud ecosystems, limiting interoperability and reducing flexibility when integrating heterogeneous sensors or custom telemetry workflows [5]. Relatively few studies describe complete open-source implementations combining embedded firmware customization, locally integrated environmental sensing, LoRa mesh communication, telemetry routing, and cloud-based data aggregation within a unified agricultural monitoring framework. Moreover, many existing agricultural monitoring platforms rely on multiple wireless layers between sensors and acquisition devices, increasing hardware complexity, energy consumption and deployment costs. In contrast, directly interfacing environmental sensors through wired ADC and I2C communication buses to embedded LoRa telemetry nodes can simplify deployment while improving reliability and reducing communication overhead.

Compared to existing agricultural LoRa/LoRaWAN platforms such as LoRaFarM [7], as well as commercial ecosystems such as Milesight Smart Agriculture, Seeed Studio SenseCAP, and Libelium Smart Agriculture, the proposed system places greater emphasis on the integration of the complete sensing-to-cloud workflow within a modular and customizable architecture. While these solutions provide mature combinations of field sensing, long-range connectivity, gateway infrastructure, and cloud-based monitoring, the proposed platform combines heterogeneous environmental sensing, embedded telemetry generation, Fog-level persistent buffering and data transfer, and domain-oriented cloud microservices using commercially available embedded hardware and open-source software components. This design provides greater flexibility in sensor selection, local data handling, and cloud-side processing, while potentially reducing the initial hardware and infrastructure cost and allowing the system to be expanded gradually according to the requirements of the agricultural deployment.

Within this context, the present paper proposes a low-cost agricultural monitoring platform based on embedded IoT sensing, LoRa mesh communication, and an Edge–Fog–Cloud architecture designed for real-time environmental telemetry acquisition and transmission. The proposed system extends the open-source Meshtastic framework through custom telemetry modules developed for agricultural monitoring applications, integrating environmental sensing, telemetry packet generation, router-based forwarding mechanisms, and power-aware communication scheduling. The platform combines embedded LoRa telemetry nodes built around the Heltec Mesh Node T114 hardware platform with intermediate routing devices and cloud-based microservices responsible for data aggregation and processing. Environmental monitoring is achieved through the integration of locally connected BME280 environmental sensors, capacitive soil moisture sensors, and TEMT6000 light sensors interfaced through I2C and ADC communication buses. Additional device-level telemetry metrics such as battery voltage, channel utilization, transmission statistics, relay counters, and uptime information are used to evaluate communication reliability and low-power operation during agricultural deployments [5,6].

The main contributions of this research can be summarized as follows: (1) the design and implementation of a low-cost embedded agricultural monitoring platform based on LoRa mesh communication and open-source firmware; (2) the development of custom telemetry modules extending the Meshtastic framework for environmental sensing, telemetry generation, and packet forwarding; (3) the integration of an Edge–Fog–Cloud architecture combining embedded sensing, intermediate routing, and cloud-based telemetry processing; and (4) a proof-of-concept experimental evaluation of the end-to-end platform operation, including environmental sensing, telemetry transmission, gateway forwarding, and cloud-side data processing under the tested sensing-node-to-router configuration.

The remainder of the paper is organized as follows. Section 2 reviews recent research in LoRa-based agricultural monitoring and embedded telemetry systems. Section 3 presents the proposed system architecture together with the embedded hardware design. Section 4 describes the firmware architecture, telemetry workflow, and LoRa communication mechanisms. Section 5 examines the cloud microservice layer through the Weather & Soil service. Section 6 discusses experimental deployment and evaluation results. Finally, Section 7 concludes the paper and outlines future research directions.

## 2. Related Work

The rapid development of precision agriculture technologies has accelerated the adoption of Internet of Things (IoT) infrastructures capable of supporting continuous environmental monitoring, automated telemetry acquisition, and data-driven agricultural management [1,2,3,4]. In recent years, multiple agricultural monitoring platforms integrating environmental sensors, wireless communication technologies and cloud services have been proposed for applications such as irrigation management, crop monitoring, greenhouse automation, and environmental data acquisition [1,2,3,4,7,8,9]. The increasing availability of low-cost embedded hardware platforms and wireless communication modules has further contributed to the development of distributed agricultural telemetry systems capable of operating in large-scale and resource-constrained environments.

Among the communication technologies used in agricultural IoT deployments, Low-Power Wide-Area Networks (LPWANs) have gained significant attention due to their ability to provide long-range communication while maintaining low energy consumption [5,6]. In particular, LoRa and LoRaWAN have become widely adopted in agricultural monitoring applications because they enable low-cost telemetry transmission over several kilometers while supporting battery-powered operation for extended periods [5,6,7,8,9]. Compared to traditional wireless communication technologies such as Wi-Fi, Bluetooth, or cellular networks, LoRa-based systems provide significantly improved communication coverage while maintaining reduced energy requirements suitable for battery-powered embedded devices operating in remote agricultural environments [6,7,8].

Following the comparison presented in Table 1 [1], LoRa communication technologies provide an advantageous balance between communication range, energy efficiency and deployment costs, making them particularly suitable for agricultural telemetry applications operating in remote or infrastructure-constrained environments [5,6,7,8,9].

Although LoRa offers lower data rates compared to Wi-Fi or Bluetooth, agricultural monitoring systems generally transmit relatively small telemetry payloads at periodic intervals, making low-bandwidth communication acceptable for most sensing applications. In addition, the reduced power consumption of LoRa-based embedded systems enables long-term battery-powered operation, which is a critical requirement for open-field agricultural deployments.

Table 1 compares the fundamental characteristics of the most widely used wireless communication technologies. The network architecture adopted by each technology also has a significant impact on the scalability and deployment cost of agricultural monitoring systems. Large-scale agricultural environments require communication infrastructures capable of covering extended areas while minimizing both hardware complexity and energy consumption. Consequently, beyond the characteristics of the physical communication technology, the network topology becomes a key design criterion for precision agriculture deployments.

**Figure 1:**
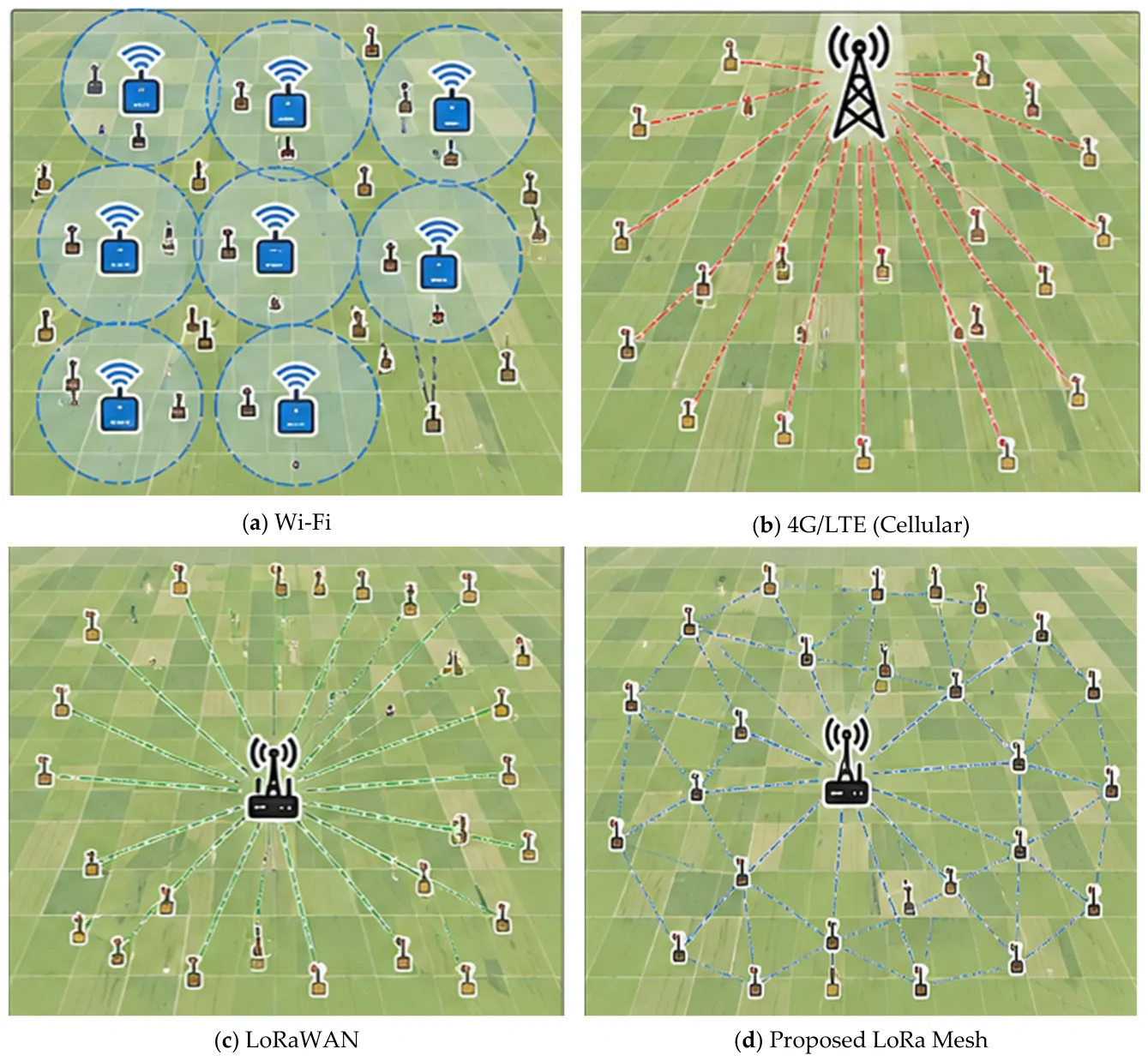
Comparison of wireless communication architectures for large-scale agricultural monitoring: (**a**) Wi-Fi infrastructure, (**b**) Cellular (4G/LTE), (**c**) LoRaWAN star topology, and (**d**) the proposed Meshtastic LoRa Mesh architecture.

As illustrated in Figure 1, infrastructure-based wireless technologies such as Wi-Fi and cellular networks require either multiple access points or individual cellular subscriptions for each sensing node, considerably increasing deployment complexity and operational costs. LoRaWAN significantly reduces infrastructure requirements by enabling long-range communication through a single gateway; yet all sensing nodes must stay within the direct communication range of the gateway. In contrast, the proposed LoRa Mesh architecture extends network coverage through multi-hop communication, allowing packets to be relayed between neighboring nodes before reaching the router/gateway. This approach can improve communication coverage and robustness while reducing dependence on direct gateway connectivity [14,15,16].

However, multi-hop communication also introduces several trade-offs. Forwarding packets through intermediate nodes can increase end-to-end delay and generate additional relay traffic, while relay activity may increase the energy consumption of intermediate devices. In larger deployments, the additional traffic may also contribute to higher channel occupancy and contention. These effects depend on the number and placement of nodes, traffic load, and radio conditions, and should therefore be considered when dimensioning larger agricultural monitoring networks [14,15,16].

Several recent studies proposed LoRa-enabled agricultural monitoring systems integrating environmental sensing devices, telemetry acquisition mechanisms, and cloud-based monitoring services [14]. Existing research demonstrated the suitability of LoRa-based architectures for applications such as irrigation management, environmental monitoring and distributed telemetry collection in open-field agricultural deployments. In parallel, multiple studies investigated LoRa communication performance in rural environments, evaluating metrics such as communication range, signal propagation, packet delivery ratio, and energy consumption under different deployment conditions [15]. Recent developments in LoRa mesh communication and telemetry routing systems further highlighted the potential of decentralized multi-hop communication mechanisms capable of improving network coverage and communication reliability in remote areas [15,16].

Environmental monitoring remains one of the most important components of smart agriculture systems due to its direct impact on crop development, irrigation management, and resource optimization [1,2,3,4]. Modern agricultural telemetry platforms frequently integrate environmental sensors for monitoring soil moisture, temperature, humidity, atmospheric pressure, luminosity, and other agronomic parameters required for precision agriculture applications. Existing studies demonstrate that embedded environmental sensing platforms commonly rely on low-cost digital and analog sensors interfaced through protocols such as I2C, SPI, and ADC communication buses.

As illustrated in Table 2, agricultural monitoring systems integrate heterogeneous environmental sensors using both analog and digital communication interfaces. Low-cost sensors such as BME280, TEMT6000, and capacitive soil moisture sensors have become increasingly popular in embedded agricultural applications due to their low power requirements, reduced hardware complexity, and compatibility with embedded telemetry platforms [1,3,7,8]. Direct integration of sensors through wired interfaces reduces communication overhead and simplifies deployment compared to architectures relying on multiple wireless sensing layers between sensors and acquisition devices.

At the same time, the increasing volume of telemetry data generated by agricultural monitoring systems has accelerated the adoption of distributed computing architectures capable of supporting scalable data acquisition, preprocessing, storage, and analysis [10,11,12,13]. Traditional cloud-centric architectures frequently rely on transmitting raw telemetry data directly toward centralized cloud infrastructures, which may generate communication overhead, increased latency, and reduced reliability in agricultural environments characterized by unstable connectivity or limited bandwidth availability. As a result, recent research has increasingly focused on Edge–Fog–Cloud architectures capable of distributing telemetry processing tasks across multiple infrastructure layers [10,11,12,13].

Within such architectures, edge devices are responsible for local sensor interfacing, telemetry acquisition, and preliminary preprocessing operations, while fog-level components provide intermediate routing, buffering, aggregation, and communication management functions before telemetry data are transmitted toward cloud infrastructures [10,11,12,13]. Cloud services are subsequently used for long-term storage, visualization, analytics, and integration with external applications. This distributed approach improves scalability, reduces communication costs, and increases resilience in agricultural monitoring deployments operating across large geographical areas. In addition, edge computing architectures support more energy-efficient operation by reducing unnecessary communication overhead and enabling adaptive telemetry scheduling directly at the embedded device level [10,11,12,13].

Recent developments in low-power embedded systems have further accelerated the adoption of edge computing architectures in agricultural monitoring applications. Platforms based on microcontrollers such as ESP32, STM32, and Nordic nRF52 have become increasingly popular in environmental telemetry systems due to their reduced power consumption, integrated communication capabilities, and compatibility with analog and digital sensing interfaces [10,12,13].

In parallel, open-source firmware frameworks have enabled the development of customizable telemetry architectures adapted to different deployment scenarios and monitoring requirements. Nevertheless, many existing implementations remain limited to proof-of-concept deployments while providing limited details regarding packet-level telemetry generation, relay forwarding mechanisms, adaptive communication scheduling, and practical low-power operation within real LoRa mesh environments. Furthermore, relatively few studies describe complete open-source implementations integrating embedded firmware customization, locally connected environmental sensing, LoRa mesh communication, telemetry routing, and cloud-based data aggregation within a unified agricultural monitoring framework [17,18,19,20].

These limitations highlight the need for scalable and modular embedded telemetry architectures capable of combining low-power sensing, decentralized communication, and distributed telemetry processing within flexible agricultural monitoring and support to decision systems.

In relation to existing open-source LoRa mesh solutions, including Meshtastic, the contribution of the proposed platform lies in extending a general-purpose communication framework toward a complete agricultural monitoring workflow. The proposed implementation integrates heterogeneous environmental sensors, embedded acquisition and preprocessing, structured environmental telemetry, Fog-level persistent buffering and data transfer, and domain-oriented cloud microservices within the same architecture. While Meshtastic provides the underlying communication capabilities, the present work adds the sensing, data-management, and cloud-processing components required to transform the communication framework into an end-to-end environmental monitoring platform for agricultural applications.

## 3. System Architecture and Embedded Design

### 3.1. System Architecture

The proposed monitoring platform uses a hierarchical Edge–Fog–Cloud architecture to collect environmental data over long distances with low power consumption. The Edge layer consists of LoRa sensing nodes distributed across the monitored agricultural area. Each node reads data from its locally connected sensors, converts the raw signals into engineering values, performs basic range checks, assembles the telemetry message, and sends it through the LoRa Mesh network.

**Figure 2:**
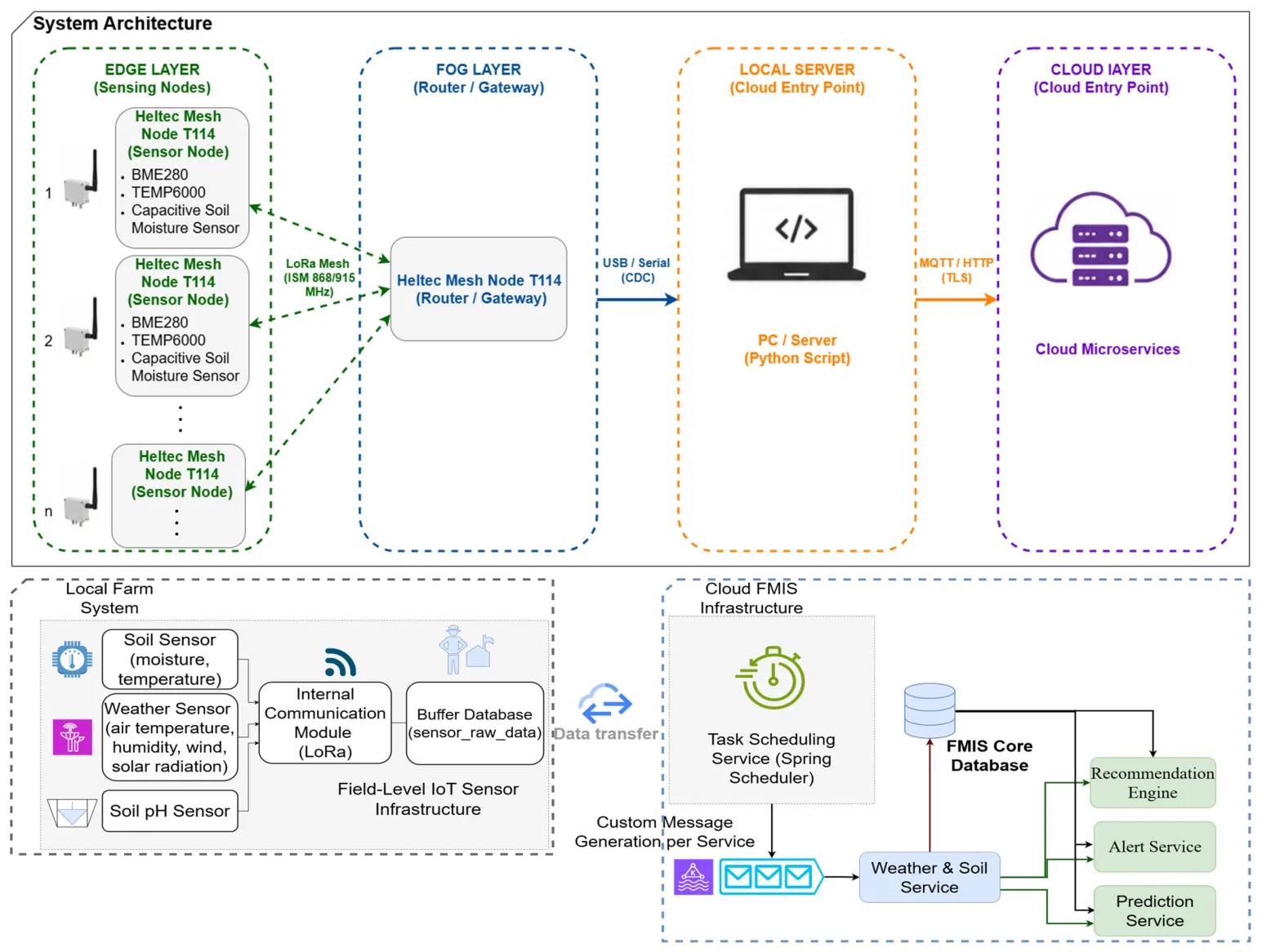
Complementary views of the proposed low-cost FMIS system architecture: (**above**) physical multipoint data acquisition and communication architecture; (**below**) logical data flow, buffering, scheduling, and cloud microservices integration.

At the Fog layer, the LoRa Mesh gateway receives the packets and forwards them to the local server. There, the packets are decoded and reconstructed as measurement records. The server checks their format and mandatory fields, verifies the timestamps, detects duplicate transmissions, and applies basic plausibility rules. It also converts the data into the common representation expected by the cloud interface. Accepted records are stored in a persistent local buffer, where they remain until their transfer to the cloud has been confirmed, Figure 2 [10,11,12,13,17,20].

A transfer component periodically reads the pending records from the local buffer and publishes them through the MQTT broker (RabbitMQ 3.13.7, was used as the MQTT broker) to the cloud ingestion component. The broker-based interface separates field-data collection from cloud-side processing, while the local buffer retains records that could not be transferred successfully and makes them available for a later retry. Once publication has been confirmed, the corresponding local records are marked as transferred [10,11,13,17,20].

On the cloud side, the ingestion component consumes the incoming messages and stores them as hourly observations. Their subsequent processing is coordinated by the Scheduler service. For each completed processing interval, the Scheduler creates a service-specific request and places it in the Weather & Soil processing queue. The request identifies the time interval and the farm or monitoring units that must be processed. The Weather & Soil service consumes the request when it becomes available and reports the outcome of the processing operation to the Scheduler.

The Weather & Soil service has access to sensor-registration data, monitoring-cell assignments, historical observations, and measurements received from other nodes. It can therefore carry out semantic and contextual checks that are not available at the local server. The service completes data normalization, evaluates data quality, and produces temporal and spatial aggregates. The resulting environmental records are stored in the Weather & Soil database and later made available to the Alert, Prediction, and Recommendation services.

This division of responsibilities keeps the field nodes lightweight and energy-efficient. Packet handling, preliminary validation, buffering, and transfer management are performed at the Fog level, while processing that requires historical, spatial, or farm-specific context is assigned to the Cloud layer [10,11,12,13,17,20].

The data path starts at the sensor interfaces of each acquisition node. The BME280 (Bosch Sensortec GmbH, Reutlingen, Germany) provides air temperature, relative humidity, and barometric pressure through the I^2^C bus, while the TEMT6000 ambient light sensor (Vishay Semiconductors GmbH, Heilbronn, Germany) and the capacitive soil moisture sensor (DFRobot, Shanghai, China) are connected to analog inputs of the nRF52840 microcontroller (Nordic Semiconductor ASA, Trondheim, Norway). The firmware performs only the conversions needed to represent the measurements in engineering units. The resulting values are inserted into Meshtastic environmental telemetry structures, encoded using Protocol Buffers, and transmitted through the SX1262 LoRa transceiver (Semtech Corporation, Camarillo, CA, USA). More demanding operations, such as historical validation and spatial analysis, are deliberately left outside the field node.

Once transmitted, a telemetry packet travels through the LoRa Mesh network towards the router connected to the local server. The experimental setup uses a single router/gateway so that the communication performance of the sensing platform can be evaluated under controlled conditions. This option does not restrict the architecture to single-hop communication. Since packet forwarding relies on the Meshtastic mesh protocol, further router nodes can be added when field dimensions, terrain, vegetation, or radio obstructions prevent a reliable direct connection to the gateway [14,15,16].

The number and distribution of sensing nodes can be adapted to the monitoring requirements. A 100 ha field, for example, may be divided into approximately one-hectare monitoring cells, with one acquisition node assigned to each cell. This represents one possible spatial partitioning strategy for agricultural fields and would result in about 100 measurement points forwarding telemetry through the same LoRa Mesh infrastructure. Alternative partitioning strategies may be defined according to crop type, soil variability, terrain, irrigation layout, or the specific purpose of the monitoring process. The 100 ha/100-node configuration is provided only as an illustrative example of field organization and was not implemented or experimentally evaluated in the present study.

The local server provides the first processing stage between the LoRa Mesh network and the cloud infrastructure. Incoming packets are decoded and reconstructed as structured measurement records. The server verifies the packet format and mandatory fields, parses the acquisition timestamp, identifies duplicate transmissions, and applies basic plausibility rules. It also converts the measurements values into the specified field names, units, and timestamp representation expected by the cloud interface. Values that cannot be interpreted reliably are rejected or marked with an appropriate validation status.

Accepted measurement records are written to a persistent local buffer. At configured intervals, the transfer component reads the records that are still pending, groups them into batches, and publishes them to the cloud through MQTT. A record is marked as transferred only after successful delivery has been confirmed. If the communication with the cloud is interrupted, the record remains in the buffer and is included in a later transfer attempt. In this way, data acquisition can continue even when Internet connectivity is temporarily unavailable.

On the cloud side, the ingestion component receives the published measurements and stores them as hourly observations. Their subsequent processing is coordinated by the Scheduler service, which determines the time interval that is ready for processing and submits the corresponding request to the Weather & Soil service. The request identifies the processing interval and the farm or monitoring units to be included.

The Weather & Soil service carries out the processing that depends on information not available at the local server. Sensor registration data, monitoring-cell assignments, previous observations, and measurements collected from nearby nodes are used to perform semantic and contextual validation. The service completes the normalization process, assigns data-quality information, and calculates temporal and spatial aggregates. The resulting environmental records are stored in the FMIS database. The data are first saved by the Weather & Soil service and are made available to the Alert, Prediction, and Recommendation services.

Each embedded node is implemented as a modular acquisition unit that combines the Heltec Mesh Node T114, the environmental sensors, and firmware derived from the Meshtastic telemetry framework. Additional sensors can be connected through the available I^2^C, ADC, GPIO, or UART interfaces without changing the communication path between the field and cloud layers. This is useful in agricultural deployments, where the required measurements may vary with crop type, soil conditions, and irrigation practices.

Router nodes have a different role from acquisition nodes. Their main purpose is to maintain mesh connectivity and forward packets from measurement points that cannot reach the gateway directly. Depending on the site, routers may be installed at intermediate or elevated locations to reduce the effects of terrain, vegetation, and other radio obstructions. Device-level telemetry, including channel utilization, airtime usage, relay counters, packet statistics, battery voltage, and uptime, can also be collected to assess network operation during deployment.

The main advantages of the proposed architecture are modularity, low cost, low power consumption and open-source extensibility. Modularity is achieved by separating sensing, routing, local validation and cloud analytics into independent layers. Low cost results from the use of commercially available embedded boards and inexpensive environmental sensors. Low-power operation is supported by duty-cycled acquisition, compact telemetry packets and LoRa communication, which is well-suited for small payloads transmitted at periodic intervals. Finally, the use of Meshtastic and open firmware enables the customization of telemetry structures, communication behavior and sensor integration without dependence on proprietary cloud ecosystems. These characteristics make the architecture suitable for scalable agricultural monitoring deployments in rural or infrastructure-constrained environments [8,9,10,11,12,13].

### 3.2. Embedded Hardware Platform

The proposed environmental monitoring node was designed around the Heltec Mesh Node T114, a compact embedded platform specifically developed for low-power LoRa Mesh applications. The board integrates the Nordic nRF52840 system-on-chip together with the Semtech SX1262 LoRa transceiver, providing a complete hardware solution for long-range wireless sensing while maintaining low energy consumption and a compact form factor. Compared to conventional IoT development boards based on Wi-Fi microcontrollers, the T114 platform offers significantly lower power requirements and native compatibility with the open-source Meshtastic ecosystem, making it particularly suitable for battery-powered agricultural monitoring systems deployed over large geographical areas [21].

The selection of the Heltec T114 platform was motivated by several design considerations. First, the board provides all essential hardware resources required for environmental sensing, including multiple digital communication interfaces, analog acquisition channels, low-power operation modes and an integrated LoRa radio. Second, the compact hardware layout considerably simplifies the integration of external environmental sensors while reducing PCB complexity and wiring requirements. Finally, the availability of an open-source software ecosystem facilitates firmware customization and future hardware extensions without dependence on proprietary communication infrastructures, an important requirement for scalable agricultural IoT deployments [21,22].

**Figure 3:**
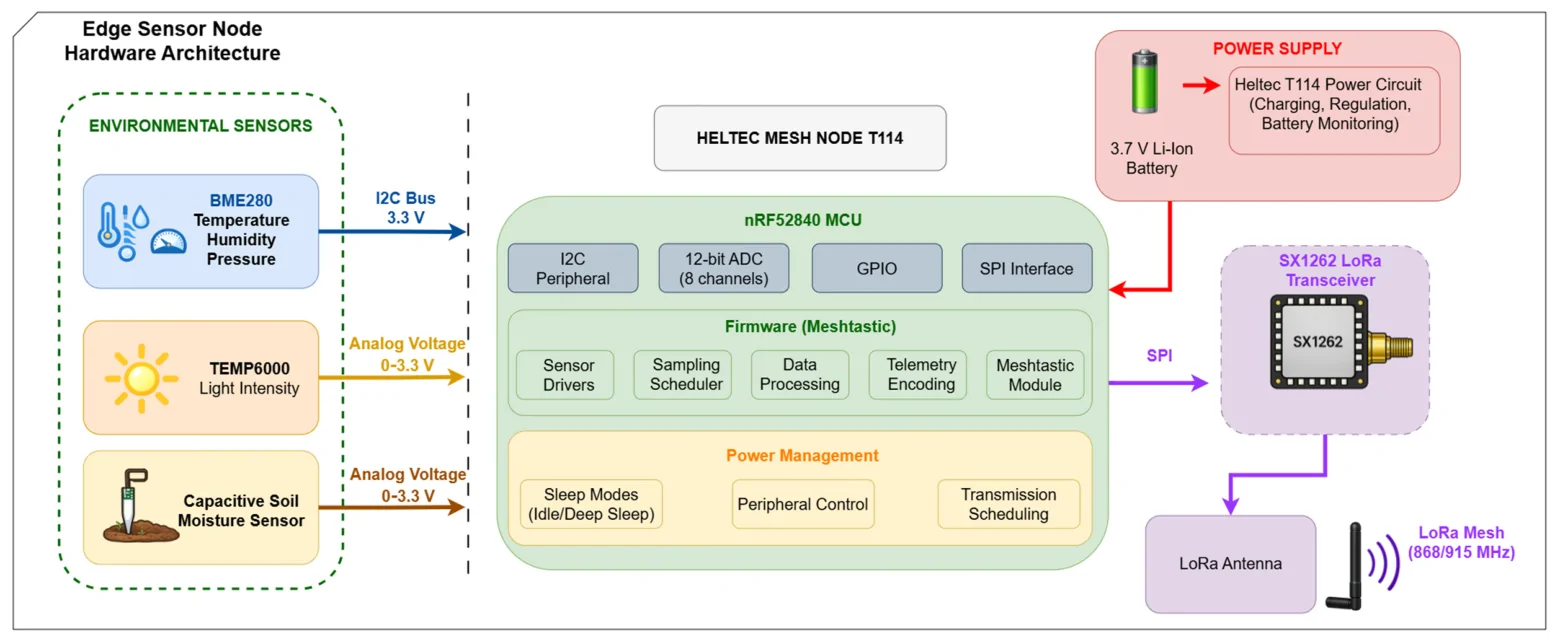
Embedded hardware architecture of the proposed edge sensing node based on the Heltec Mesh Node T114 platform.

At the core of the embedded sensing node is the Nordic nRF52840, a highly integrated System-on-Chip (SoC) specifically designed for low-power wireless embedded applications, see Figure 3. The device combines a 64 MHz Arm Cortex–M4F processor with 1 MB of Flash memory and 256 KB of RAM, providing sufficient computational resources for deterministic sensor acquisition, peripheral management and communication with the external LoRa transceiver while maintaining a very low energy footprint [22]. In addition to its processing capabilities, the nRF52840 integrates an extensive set of hardware peripherals, including multiple SPI, I^2^C (TWI), UART, PWM and GPIO interfaces, together with a 12 bit Successive Approximation Register (SAR) Analog-to-Digital Converter (SAADC). The availability of these peripherals eliminates the need for external interface controllers, reducing both hardware complexity and manufacturing cost.

The choice of the nRF52840 was primarily motivated by its balance between computational performance, peripheral flexibility and ultra-low-power operation. Unlike more resource-demanding application processors, the Cortex-M4F architecture provides sufficient processing capability for environmental monitoring while maintaining deterministic execution and predictable timing, both of which essential for periodic sensing applications. Furthermore, the floating-point unit integrated within the processor enables efficient numerical operations without significantly increasing execution time, making the platform suitable for applications involving sensor calibration, engineering unit conversion and lightweight numerical processing [22].

Long-range wireless connectivity is provided by the Semtech SX1262, which is connected to the nRF52840 through a dedicated Serial Peripheral Interface (SPI) bus, as illustrated in Figure 3. The SX1262 belongs to Semtech’s second generation of LoRa transceivers and has been optimized for battery-powered Internet of Things devices operating over sub-GHz frequency bands. The transceiver supports both LoRa^®^ and FSK modulation schemes, transmission power up to +22 dBm, receiver sensitivity down to –148 dBm and a link budget approaching 170 dB, enabling reliable communication over several kilometers under favorable propagation conditions [23].

The SPI interface was selected because of its high throughput, deterministic timing and full-duplex operation, allowing rapid configuration of the radio registers and efficient transfer of telemetry payloads between the microcontroller and the transceiver. Unlike shared communication buses such as I^2^C, SPI establishes a dedicated point-to-point connection between the MCU and the radio module, minimizing communication latency and preventing bus contention during packet preparation. Hardware interrupt lines (DIO) connected through the GPIO interface notify the microcontroller about transmission completion, reception events and radio status changes, allowing efficient synchronization between the processing unit and the wireless subsystem while avoiding continuous polling of the transceiver [22,23].

The environmental sensing subsystem combines both digital and analog sensors in order to acquire the principal environmental variables required for precision agriculture. This heterogeneous approach allows each sensing parameter to be acquired using the interface that best matches the electrical characteristics of the corresponding sensor, reducing hardware complexity while supporting consistent data acquisition [23].

The Bosch BME280 digital environmental sensor is responsible for measuring ambient temperature, relative humidity and atmospheric pressure. The sensor integrates factory-calibrated sensing elements and internal compensation algorithms, supporting environmental measurements with limited processing effort from the host microcontroller [24]. In the proposed hardware platform, the BME280 communicates with the nRF52840 through the I^2^C interface operating at 3.3 V. The use of I^2^C significantly reduces the number of required interconnection wires, since all environmental variables are transferred through a common two-wire serial bus. This architecture simplifies PCB routing and facilitates the future integration of additional digital sensors sharing the same communication bus [24].

In addition to the digital environmental sensor, the proposed sensing platform integrates two analog sensors dedicated to monitoring ambient illumination and soil moisture, respectively. Both devices provide analog voltage outputs that are directly sampled by the nRF52840 integrated SAADC peripheral. This design choice eliminates the need for external analog front-end circuits or dedicated sensor controllers, reducing hardware complexity, manufacturing cost, and overall power consumption while maintaining sufficient accuracy for precision agriculture applications [24].

Ambient illumination is measured using the TEMT6000 ambient light sensor. The device is based on a silicon NPN phototransistor with a spectral response closely matching that of the human eye, exhibiting maximum sensitivity around 570 nm [11]. Unlike digital luminosity sensors, the TEMT6000 generates an output current proportional to the incident light intensity, which is converted into an analog voltage through an external load resistor. The resulting voltage is directly connected to one of the analog input channels of the nRF52840, allowing the embedded platform to estimate the surrounding illumination level with minimal hardware requirements. Although the TEMT6000 is not intended for laboratory-grade photometric measurements, it provides a simple and energy-efficient solution for detecting variations in ambient light conditions that may influence crop development or indicate day–night transitions [25].

Soil water availability is monitored using a capacitive soil moisture sensor, whose operating principle is based on measuring variations in the dielectric permittivity of the surrounding soil rather than electrical conductivity. Compared to conventional resistive probes, capacitive sensors exhibit significantly improved long-term stability because the sensing electrodes are electrically isolated from the soil, reducing corrosion and minimizing measurement drift caused by electrochemical degradation [26]. Similarly to the TEMT6000, the sensor provides an analog voltage proportional to the measured capacitance, which is sampled through the integrated SAADC of the microcontroller. Since the output voltage depends on soil composition, texture, temperature, and sensor placement, a two-point calibration was performed before deployment. The dry reference was determined experimentally as a raw ADC value of approximately 350, while the wet reference was approximately 120. These values were used as the 0% and 100% endpoints, respectively, for linear normalization of the soil-moisture signal. Intermediate ADC readings were mapped between these limits and constrained to the 0–100% range. The resulting value is therefore interpreted as a normalized relative soil-moisture indicator suitable for agricultural monitoring rather than as a direct measurement of volumetric water content [26].

The proposed embedded platform intentionally combines both digital and analog communication interfaces in order to accommodate sensors with different electrical characteristics while maintaining a simple and modular hardware architecture. Figure 3 illustrates the interconnection between the sensing subsystem, the processing unit and the wireless communication module.

Digital environmental measurements are acquired through the Inter-Integrated Circuit (I^2^C) bus, which connects the BME280 sensor to the nRF52840 microcontroller. The I^2^C interface was selected because it requires only two bidirectional signal lines—Serial Data (SDA) and Serial Clock (SCL)—regardless of the number of connected devices. This considerably reduces PCB routing complexity and facilitates future expansion of the sensing platform through the addition of other compatible environmental sensors without requiring extra communication peripherals. Furthermore, the master–slave architecture of the I^2^C protocol allows the microcontroller to sequentially access individual sensor registers while ensuring reliable digital communication with minimal hardware overhead [21,22,23,24,25,26].

Unlike the BME280, both the TEMT6000 and the capacitive soil moisture sensor generate analog electrical signals. Consequently, these sensors are interfaced through the 12-bit Successive Approximation Register Analog-to-Digital Converter (SAADC) integrated within the nRF52840. The SAADC converts the analog voltages into digital values that can subsequently be interpreted by the embedded application. Integrating the analog converter within the microcontroller eliminates the need for external ADC components, thereby reducing the bill of materials, PCB area and power consumption. In addition, the relatively high resolution of the SAADC enables the detection of small variations in sensor output voltage, improving the sensitivity of illumination and soil moisture monitoring [21,22,23,24,25,26].

Communication between the microcontroller and the SX1262 radio module is implemented through a dedicated Serial Peripheral Interface (SPI). Compared to I^2^C, SPI provides significantly higher data throughput and deterministic full-duplex communication, making it particularly suitable for transferring configuration registers and radio payloads between the processing unit and the LoRa transceiver. Since the radio module operates independently from the sensing subsystem, the use of separate communication buses prevents interference between sensor acquisition and wireless communication tasks, contributing to improved system responsiveness and hardware modularity [21,22,23,24,25,26].

Several General-Purpose Input/Output (GPIO) pins are additionally employed for peripheral control and hardware synchronization. In addition to conventional digital input and output functions, GPIO lines are used to interface hardware interrupt signals generated by the SX1262 transceiver, allowing the microcontroller to detect transmission completion, packet reception events and radio status changes without continuous polling. Additional GPIO resources remain available for integrating future peripherals, enabling the hardware platform to accommodate further sensing capabilities while preserving its modular design philosophy [21,22,23,24,25,26].

The proposed sensing node is powered by a rechargeable 3.7 V lithium-ion battery, enabling autonomous operation in outdoor agricultural environments where continuous access to the electrical grid is unavailable. As illustrated in Figure 3, the battery is connected directly to the onboard power-management circuitry of the Heltec Mesh Node T114, which integrates battery charging, voltage regulation and protection mechanisms required for safe long-term operation. The regulated 3.3 V supply voltage powers both the nRF52840 microcontroller and the connected environmental sensors, ensuring stable operation despite gradual battery voltage variations during charge and discharge cycles [21,22,23,24,25,26].

An important design objective was to minimize the number of external power-management components. The T114 platform integrates the circuitry required for battery charging and voltage regulation, allowing the sensing node to be implemented without additional power conditioning modules. This significantly reduces PCB complexity, manufacturing cost and assembly effort while improving system reliability. Moreover, the common 3.3 V power rail simplifies the integration of both digital and analog peripherals, since the BME280, TEMT6000 and capacitive soil moisture sensor are all compatible with this operating voltage [21,22,23,24,25,26].

Battery status monitoring represents another essential hardware feature for autonomous environmental monitoring systems. The battery voltage is continuously monitored through dedicated circuitry available on the T114 platform, allowing the embedded application to estimate the remaining energy capacity and report battery status together with environmental measurements. Continuous monitoring of the power source facilitates preventive maintenance and enables the identification of nodes requiring battery replacement or recharging before communication failures occur. This capability becomes increasingly important in large-scale agricultural deployments, where manually inspecting every sensing node would be impractical [21,22,23,24,25,26].

Low energy consumption was considered throughout the hardware design process in order to maximize node autonomy during long-term field deployment. Rather than relying on a single ultra-low-power component, the proposed platform combines several energy-efficient devices whose characteristics complement each other. The nRF52840 microcontroller incorporates multiple low-power operating modes and an optimized peripheral architecture, while the SX1262 transceiver has been specifically developed for long-range communication with extremely low receive and standby currents. Similarly, the BME280 environmental sensor provides microampere-level current consumption together with an ultra-low-power sleep mode, making it particularly suitable for periodic environmental monitoring applications [21,22,23,24,25,26].

Another important aspect concerns the selection of communication interfaces. The use of I^2^C for digital sensing minimizes the number of required interconnection lines, thereby reducing both hardware complexity and leakage currents associated with additional interface circuitry. Analog sensors are connected directly to the integrated SAADC of the microcontroller, eliminating the need for external analog-to-digital converters and reducing component count. Furthermore, the dedicated SPI interface between the nRF52840 and the SX1262 provides high-speed communication while remaining active only during radio configuration and payload transfer, resulting in negligible impact on the overall energy budget of the sensing node [21,22,23,24,25,26].

The hardware architecture was intentionally designed to remain compact, modular and easily reproducible. All sensing devices are directly connected to the embedded platform through standard communication interfaces without requiring dedicated expansion boards or complex signal-conditioning circuits. This modular organization facilitates maintenance, simplifies future sensor replacement or expansion, and allows identical acquisition nodes to be replicated across large agricultural fields with minimal hardware modifications. Consequently, the proposed embedded platform provides a balanced compromise between sensing capability, communication performance, manufacturing cost and energy efficiency, making it well suited for distributed precision agriculture applications [21,22,23,24,25,26].

The selection of the hardware components was primarily driven by the need to achieve long-term autonomous operation while maintaining reliable environmental sensing and long-range wireless communication. Table 3 summarizes the typical electrical characteristics of the principal hardware components employed in the proposed sensing node. The reported values correspond to typical operating conditions specified by the manufacturers and provide an indication of the contribution of each component to the overall energy budget of the embedded platform [21,22,23,24,25,26].

As shown in Table 3, the selected hardware components exhibit relatively low operating currents compared to conventional wireless sensing platforms. The nRF52840 and BME280 contribute only a small fraction of the total energy consumption during sensing operations, whereas the SX1262 represents the dominant energy consumer during radio transmission. Consequently, minimizing the radio duty cycle has a greater impact on overall node autonomy than reducing the sensor acquisition time.

Based on the prototype components used in this study, the indicative hardware cost of one sensing node is approximately EUR 45–65, depending on the supplier and component configuration. This estimate includes the Heltec Mesh Node T114, environmental sensors, battery, and basic interconnection components, but excludes the enclosure, installation, gateway/server infrastructure, and maintenance costs. The reported value should therefore be interpreted as a prototype-level hardware estimate rather than as the total cost of a complete field deployment.

Based on the proposed hardware architecture, the embedded firmware implements periodic sensor acquisition, environmental telemetry generation and LoRa Mesh communication using the Meshtastic framework. The software organization, telemetry workflow and communication mechanisms are described in detail in the following section.

## 4. Firmware Architecture and LoRa Communication

The firmware of the proposed environmental monitoring platform was developed as an extension of the open-source Meshtastic framework running on the Heltec Mesh Node T114 platform. Instead of implementing a proprietary communication protocol, the proposed solution reuses the native Meshtastic networking stack while extending the environmental telemetry subsystem to acquire measurements from both digital and analog sensors. This approach preserves compatibility with the existing Meshtastic ecosystem, including gateways and client applications, while enabling the transmission of agricultural telemetry without modifying the underlying mesh communication protocol.

The proposed firmware builds on the existing Meshtastic codebase but includes several platform-specific adaptations. Core communication services and data structures provided by Meshtastic are retained as the basis of the communication subsystem, while the environmental telemetry workflow was customized for agricultural monitoring. The modifications introduced in this work include the integration of the BME280, TEMT6000, and capacitive soil-moisture sensors, analog and digital data acquisition, measurement preprocessing and normalization, soil-moisture calibration, adaptation of the environmental telemetry fields, and forwarding of the resulting measurements toward the local processing infrastructure. Thus, the implementation combines native Meshtastic functionality with modified and newly introduced components required by the proposed sensing-to-cloud workflow.

The implemented firmware follows a modular organization in which sensor acquisition, telemetry generation and wireless communication are handled by independent software components. Environmental measurements are periodically acquired from the BME280, TEMT6000, and capacitive soil moisture sensors, converted into engineering units, and mapped to the standard EnvironmentMetrics telemetry structure. The resulting telemetry object is subsequently serialized using Protocol Buffers and forwarded to the Meshtastic networking layer, where it is encapsulated into a MeshPacket before LoRa transmission [27,28,29].

From an architectural perspective, the firmware can be divided into two complementary functional layers. The first layer implements the complete telemetry workflow, including hardware initialization, sensor acquisition, local preprocessing, validation, telemetry construction, and Protocol Buffers serialization. The second layer relies on the native Meshtastic communication stack to manage packet scheduling, LoRa transmission, mesh routing, acknowledgements, and packet retransmissions. This separation simplifies firmware maintenance while allowing sensing algorithms and communication services to evolve independently.

### 4.1. Environmental Telemetry Workflow

**Figure 4:**
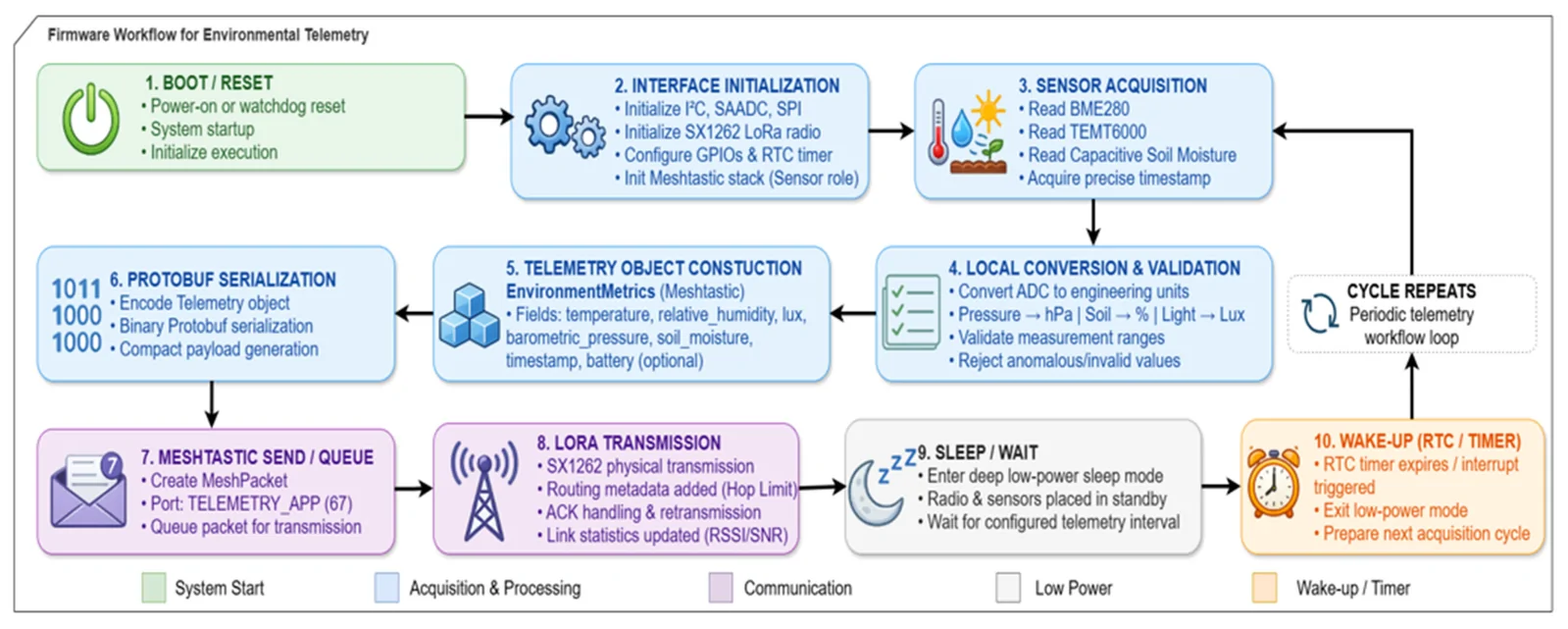
Firmware state machine implemented by the environmental sensing node.

The environmental telemetry workflow implemented by the proposed firmware is illustrated in Figure 4. The workflow follows a periodic sequential execution model in which the sensing node periodically wakes up, initializes the required hardware resources, acquires environmental measurements, performs lightweight preprocessing, constructs a telemetry message, serializes the data using Protocol Buffers, and forwards the resulting packet to the Meshtastic communication layer for wireless transmission. After completing the transmission, the node returns to a low-power waiting state until the next acquisition interval expires. This cyclic execution model reduces processor activity while supporting periodic environmental monitoring.

Following power-up or system reset, the firmware performs the initialization of all hardware peripherals required by the sensing application. The I^2^C interface is configured for communication with the BME280 environmental sensor, while the SAADC peripheral is initialized to acquire analog measurements from the TEMT6000 light sensor and the capacitive soil moisture sensor. In parallel, the SPI interface establishes communication with the SX1262 LoRa transceiver, and the Meshtastic framework initializes the networking services required for packet transmission. Successful completion of this stage prepares the sensing node for subsequent periodic acquisition and transmission cycles.

Once the hardware peripherals become operational, the firmware sequentially acquires measurements from all connected environmental sensors. Digital measurements, including temperature, relative humidity and atmospheric pressure, are obtained through the I^2^C interface, whereas ambient light intensity and soil moisture are sampled through dedicated SAADC channels. Acquiring all measurements during the same execution cycle ensures temporal consistency between the reported environmental parameters while maintaining a predictable execution time.

Raw sensor measurements are subsequently converted into engineering units before transmission. Atmospheric pressure is converted from pascals to hectopascals, while the analog outputs of the illumination and soil moisture sensors are transformed into normalized physical quantities using predefined calibration parameters. During the same stage, the firmware performs lightweight validation by rejecting physically unrealistic values and limiting measurements to predefined operating ranges. Keeping the preprocessing stage intentionally simple reduces computational overhead while preventing corrupted measurements from being transmitted through the LoRa network.

After local preprocessing, the validated measurements are mapped directly to the native Meshtastic EnvironmentMetrics structure. Temperature, relative humidity, atmospheric pressure, ambient illumination and soil moisture are assigned to their corresponding telemetry fields, preserving compatibility with the standard Meshtastic telemetry schema. Reusing the native telemetry object eliminates the need for proprietary application payloads while enabling interoperability with existing Meshtastic gateways and monitoring applications.

Once the telemetry object has been populated, the firmware serializes the message using Google’s Protocol Buffers binary encoding. Compared with text-based formats such as JSON or XML, Protocol Buffers significantly reduce payload size while providing deterministic encoding and efficient parsing. These characteristics are particularly important for LoRa communication, where the available payload capacity is limited and minimizing airtime directly contributes to lower energy consumption and improved channel utilization [30].

Following serialization, the telemetry payload is forwarded to the Meshtastic communication layer, where it is encapsulated into a MeshPacket together with the routing metadata required by the mesh protocol. The networking stack subsequently schedules the transmission according to the configured radio parameters and network state before forwarding the packet to the SX1262 LoRa transceiver. This separation between telemetry generation and packet transmission allows the sensing application to remain independent of the underlying mesh communication mechanisms.

After the transmission process has completed, the sensing node returns to a low-power waiting state until the next scheduled telemetry interval expires. This wake–acquire–process–transmit–sleep execution model significantly reduces processor utilization and radio activity, thereby extending battery lifetime during long-term agricultural deployments [28,30].

### 4.2. LoRa Mesh Communication

The proposed communication subsystem is implemented on top of the native Meshtastic networking framework using the SX1262 LoRa transceiver and the RadioLib abstraction layer. Unlike conventional point-to-point LoRa communication, the implemented solution relies on the Meshtastic mesh protocol to transport environmental telemetry generated by multiple acquisition nodes toward a single router node acting as the gateway. Each sensing node periodically generates a telemetry packet containing the environmental measurements described in Section 4.1 and forwards it to the communication layer, where the packet is encapsulated into a MeshPacket structure before wireless transmission. This architecture allows the sensing application to remain independent of the underlying radio implementation while preserving compatibility with the Meshtastic ecosystem [23,27,28,29].

The LoRa transceiver is initialized through the RadioLib interface, which abstracts the low-level interaction with the SX1262 radio and prepares the communication subsystem for continuous operation. During the initialization stage, the firmware configures the radio driver, enables packet reception, and initializes the Meshtastic networking services responsible for packet management and routing. The communication parameters are provided by the Meshtastic configuration layer, allowing the sensing application to remain independent of the underlying radio implementation [27,28,30].

**Figure 5:**
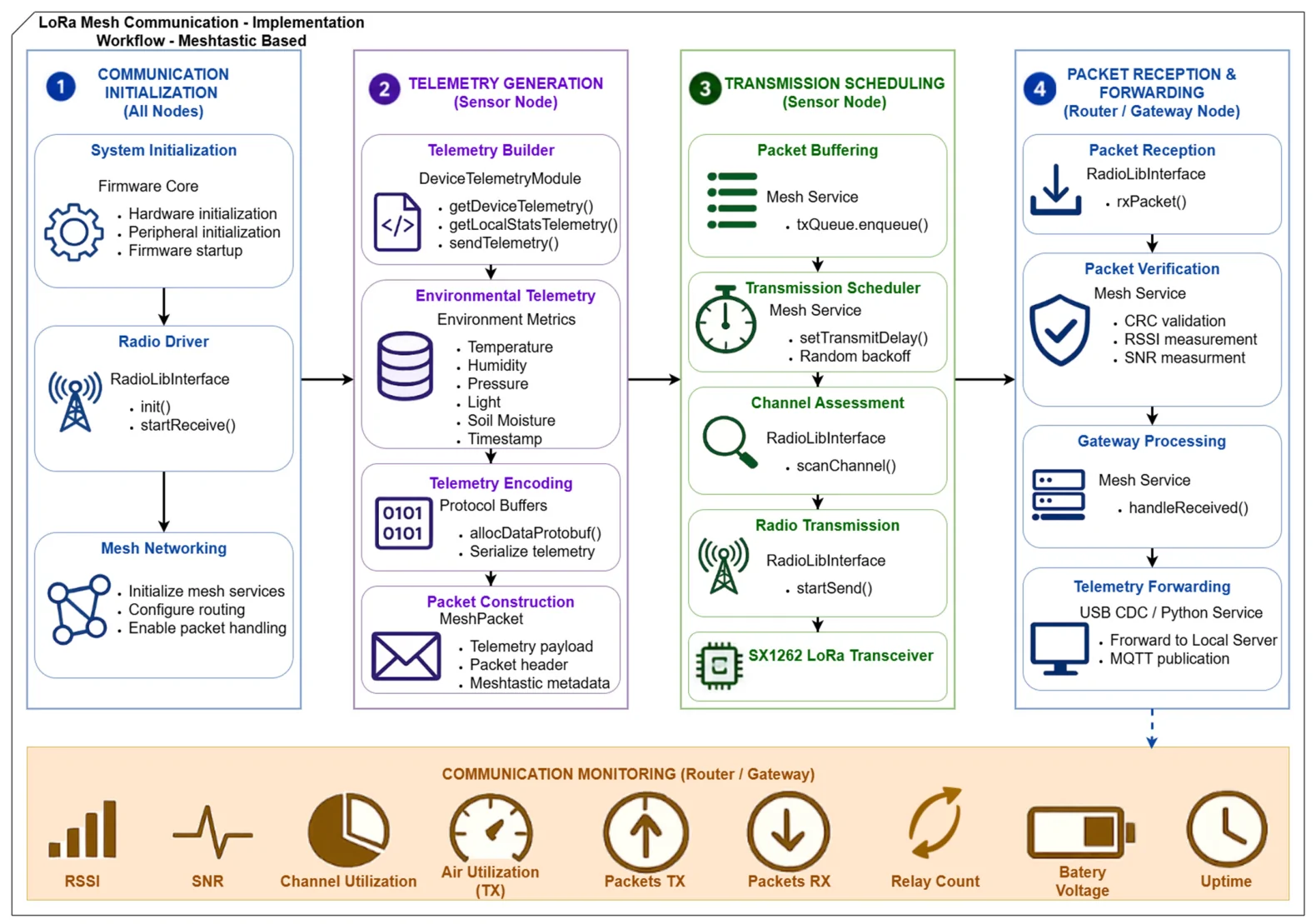
LoRa Mesh firmware architecture and communication workflow based on the Meshtastic framework.

The proposed implementation follows a modular software organization in which each communication stage is encapsulated within a dedicated firmware component. As shown in Figure 5, communication initialization, telemetry generation, transmission scheduling, and packet reception are implemented as independent processing stages that exchange standardized Meshtastic data structures.

This modular organization simplifies firmware maintenance, facilitates future extension of sensing capabilities and allows the communication mechanisms to evolve indepen-dently of the environmental acquisition modules [29].

As illustrated in Figure 5, the communication workflow is clearly separated into four consecutive stages:

1. First, the communication subsystem initializes the RadioLib driver together with the Meshtastic networking services responsible for packet management and radio operation;
2. Once environmental telemetry has been generated by the sensing application, the measurements are encapsulated into Meshtastic telemetry objects and serialized using Protocol Buffers before being encapsulated into Meshtastic MeshPacket structures;
3. The communication layer subsequently schedules packet transmission through the internal transmission queue, applies collision avoidance mechanisms based on randomized backoff and Channel Activity Detection (CAD);
4. And finally it forwards the packet to the SX1262 transceiver for wireless transmission. At the router node, incoming packets are validated, communication statistics are updated, and the recovered telemetry is forwarded through the USB CDC interface to the local server, where higher-level processing is performed.

**Figure 6:**
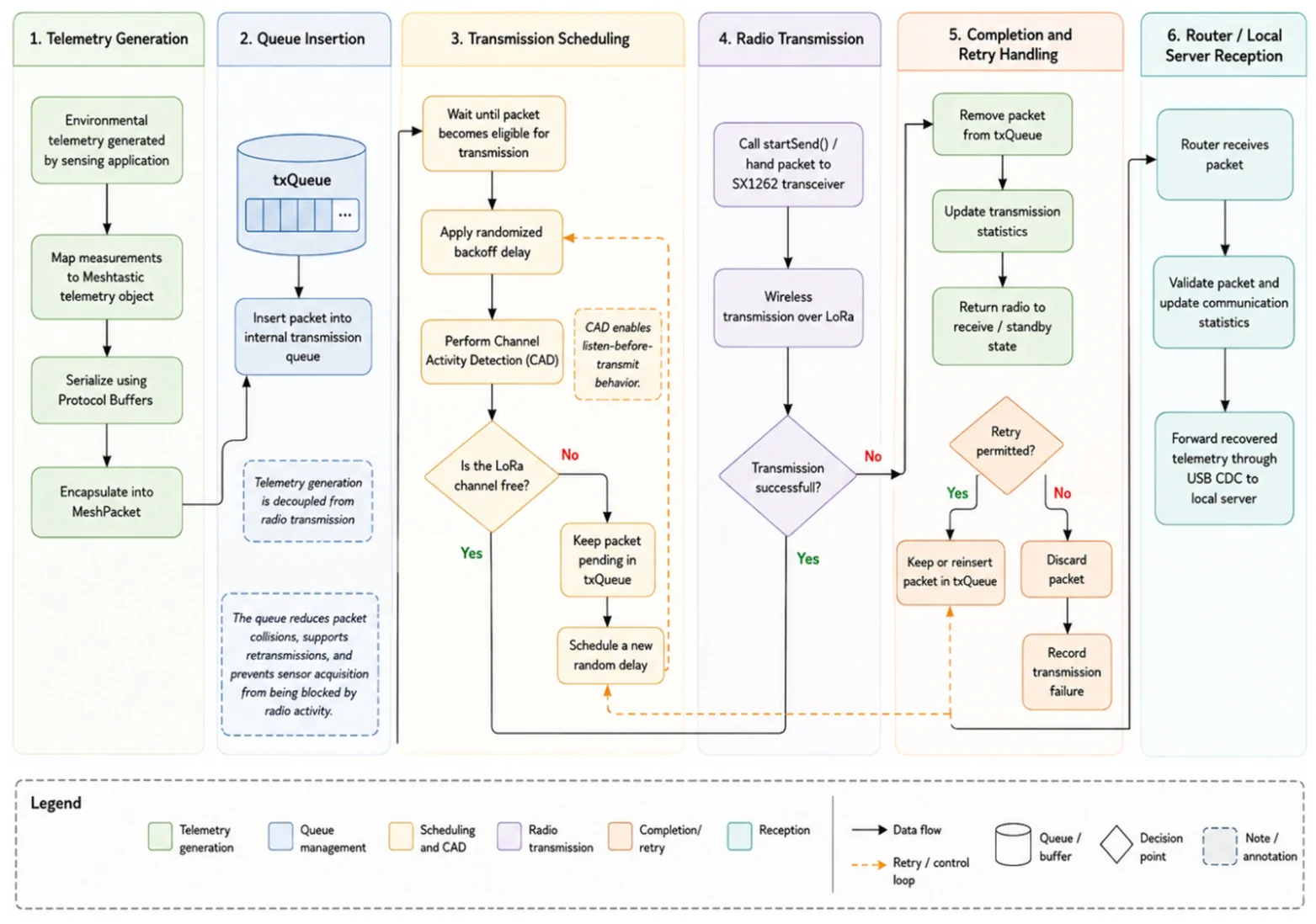
Internal transmission-queue scheduling and packet-forwarding workflow of the Meshtastic-based sensing platform.

Figure 5 provides a high-level view of the communication path, from telemetry generation to packet reception at the router. The internal operation of the transmission queue, including packet insertion, randomized backoff, Channel Activity Detection, radio transmission, retry handling, and forwarding to the local server, is detailed in Figure 6.

Rather than transmitting packets immediately after telemetry generation, the proposed implementation introduces a transmission scheduling mechanism based on an internal transmission queue. Every telemetry packet is first inserted into the transmission queue (txQueue), where it remains pending until the communication subsystem determines that transmission can safely begin. This decoupling between telemetry generation and radio transmission prevents blocking the sensing application and enables the communication stack to manage packet scheduling independently of sensor acquisition. Furthermore, the queue mechanism facilitates retransmissions and reduces the probability of packet loss when multiple nodes attempt to access the wireless medium simultaneously [28].

An important design aspect of the proposed implementation is the separation between the sensing application and the communication subsystem. Environmental acquisition modules generate telemetry independently of the radio state, while the communication layer autonomously manages packet buffering, transmission scheduling, channel access, and packet forwarding. Consequently, sensor acquisition is never blocked by ongoing radio activity, allowing deterministic sampling intervals to be maintained even when channel occupancy increases. This modular organization also facilitates future extensions of the sensing platform without requiring modifications to the underlying communication stack.

To reduce packet collisions within the shared wireless channel, the firmware employs a randomized transmission scheduling strategy. Before every transmission, a random backoff interval is introduced through the setTransmitDelay() function, allowing neighboring nodes to defer their transmissions by different time intervals. This mechanism minimizes the probability that multiple sensing nodes initiate radio transmission simultaneously, thereby reducing channel contention and improving overall communication reliability in dense deployments. Unlike fixed transmission intervals, the randomized scheduling strategy distributes channel access more uniformly among the participating nodes [28].

Before initiating the actual radio transmission, the firmware performs Channel Activity Detection (CAD) using the hardware capabilities of the SX1262 transceiver. The communication layer scans the current radio channel to determine whether another LoRa transmission is already in progress. If channel activity is detected, the node immediately returns to the receive state and postpones transmission by scheduling a new random delay. Only when the channel is considered free does the firmware invoke the startSend() routine, which transfers the packet to the SX1262 transceiver for wireless transmission. This listen-before-transmit strategy significantly reduces the likelihood of packet collisions and contributes to more efficient channel utilization without requiring centralized medium access control [23,31].

The experimental deployment consisted of one environmental sensing node communicating with a single router node that simultaneously performed the gateway function. The sensing node periodically transmitted environmental telemetry through a direct LoRa radio link, while the router received the incoming packets and forwarded them to the local server for subsequent validation and MQTT publication. Consequently, the present experimental configuration did not evaluate true multi-hop mesh operation. The experiment was intended to validate the sensing-to-gateway communication path and the end-to-end integration of the proposed platform. The communication framework remains compatible with the native Meshtastic multi-hop mechanisms. Future experimental work will extend the current setup to multiple sensing and router nodes, considering both direct node-to-gateway communication and true multi-hop mesh configurations over larger distances. These experiments will be used to evaluate packet loss, packet-delivery performance, end-to-end transmission time, retransmission behavior, and the impact of increasing node count and communication distance on network performance [31].

In addition to environmental telemetry, the firmware continuously collects communication statistics exposed by the Meshtastic framework. These include received signal strength (RSSI), signal-to-noise ratio (SNR), channel utilization, transmission airtime utilization (air_util_tx), transmitted and received packet counters, relay operations, battery voltage, and node uptime. These metrics provide continuous insight into the operating status of the communication subsystem and the deployed nodes and were used to characterize communication behavior during the experimental evaluation [31].

In addition to environmental telemetry, the firmware continuously collects communication statistics exposed by the Meshtastic framework. These include received signal strength (RSSI), signal-to-noise ratio (SNR), channel utilization, transmission airtime utilization (air_util_tx), transmitted and received packet counters, relay operations, battery voltage, and node uptime. These metrics provide continuous insight into the operating status of both the communication subsystem and the deployed sensing nodes, allowing network behavior and communication performance to be monitored throughout long-term field deployments [31].

## 5. Cloud Microservices—Weather and Soil Microservice

The cloud layer of the proposed platform is organized as a set of domain-oriented microservices. Among these components, the Weather & Soil service is responsible for transforming the measurements received from the local farm infrastructure into consistent environmental information that can be used by the Alert, Prediction, and Recommendation services. Its role is therefore broader than simple cloud storage. The service manages the environmental-data processing workflow, maintains the corresponding cloud database tables, and exposes processed records to the other FMIS components.

Measurements arriving from the Fog layer have already passed a preliminary processing stage. The local server verifies the packet structure and mandatory fields, parses the acquisition timestamp, detects duplicate transmissions, applies basic plausibility rules, and converts the decoded telemetry into the common representation accepted by the cloud interface. These checks are intended to identify malformed or unusable messages before transmission. They do not replace the domain-level validation performed in the Cloud [10,11,12,13,17,20].

After cloud ingestion, each environmental record received from the Fog layer is stored in the *sensor_data* table as the original Cloud input. Its payload and reception metadata are retained so that the record can be traced back to the corresponding sensing node and, when required, reprocessed using updated validation rules. The Weather & Soil service then performs data validation and normalization, followed by temporal alignment and spatial association. The accepted measurements are subsequently kept in the *weather_data* and *soil_data* tables.

In the proposed system, an environmental record is a structured telemetry entry built from measurements received by the local server and kept in the Fog-level buffer database. It represents a single acquisition node at a particular measurement time, combining the environmental values reported by that node with the identifiers and timestamps needed for later processing. Pending records are retrieved from the local buffer by the Scheduler and sent to the Weather & Soil service.

The environmental record is not the same as the compact telemetry packet sent across the LoRa Mesh network. Encoded with Protocol Buffers, the packet is a communication-level structure: it carries the sensor readings along with the information needed for transmission and routing. Once it arrives at the local server, the packet is decoded, validated, and rebuilt as an environmental record. That record follows a common transfer representation, allowing it to be stored in the buffer database and processed later without depending on the original Meshtastic packet format.

In the reference configuration, each sensing node creates one environmental record every hour. This record retains the individual measurements collected during the sampling cycle rather than statistical values derived from multiple samples. The environmental payload covers air temperature, relative humidity, atmospheric pressure, ambient illuminance, and normalized soil moisture. Alongside these measurements are the source-node identifier, acquisition timestamp, matching farm and field identifiers, and the processing status needed by the local store-and-forward mechanism [24,25,26].

The fields required to identify, transfer, and process an hourly environmental record are summarized in Table 4. The structure contains a common metadata section and an environmental payload. The metadata identifies the source node, monitoring location, and acquisition time, while the payload contains the measurements acquired during the corresponding sampling cycle. Transfer-related fields are maintained by the local store-and-forward mechanism and updated as the record moves through the Fog-to-Cloud workflow.

The logical fields presented in Table 4 are serialized using the common JSON representation illustrated in Listing 1. Environmental measurements are grouped in the payload object, while node identification, acquisition timing, and transfer-control information are retained at the top level.

At configured intervals, the Cloud-based Scheduler initiates the transfer of pending environmental records from the Fog-level buffer. Records marked as PENDING are selected, grouped into batches, and published to the Cloud ingestion component through the configured MQTT interface. The records remain stored in the local buffer until successful reception is acknowledged by the Cloud. After confirmation, their transfer status is updated accordingly; if the transfer fails, the records remain available for a subsequent retry.

Once the records are accepted by the Cloud ingestion component and stored in the sensor_data table, the Scheduler coordinates their subsequent processing. For each processing interval, it creates a corresponding execution in the ws_processing_runs table, identifies the records associated with that execution, and submits them to the Weather & Soil service. The Scheduler therefore coordinates both the transfer cycle and the subsequent Cloud-side processing, while the Fog-level buffer provides persistent storage until successful delivery is confirmed.


**Listing 1.** Example JSON serialization of a pending hourly environmental record stored in the Fog-level buffer.{               “recordId”: “ENV-JSH6UGB4AZ-20250618T100000Z”,                “recordType”: “ENVIRONMENTAL”,                “sourceNodeId”: “JSH6UGB4AZ”,                “farmId”: 1,                “fieldId”: 4,                “monitoringCellId”: “CELL-017”,                “observedAt”: “2025-06-18T10:00:00Z”,                “bufferedAt”: “2025-06-18T10:00:06Z”,                “packetSequenceNumber”: 1842,                “payload”: {                    “temperatureC”: 27.4,                    “relativeHumidityPct”: 54.2,                    “pressureHpa”: 1008.6,                    “illuminanceLx”: 718.0,                    “soilMoisturePct”: 31.7               },               “transferStatus”: “PENDING”,               “retryCount”: 0 }


The Weather & Soil service begins by validating and normalizing the incoming data. First, it confirms that the source node is registered and linked to the field and monitoring cell identified in the record. Next, the service checks whether the expected environmental fields are present and have the correct types, then applies rules specific to each parameter. Relative humidity and normalized soil moisture, for instance, must remain within the 0–100% range; illuminance cannot be negative. Temperature and atmospheric pressure, meanwhile, are evaluated against the sensors’ configured operating ranges and the environmental conditions at the deployment site.

Validation is performed independently for each measurement field. Consequently, a record can still be partially accepted when some values are valid while others are missing or outside their configured range. For example, temperature, humidity, pressure, and illuminance may be retained even if the soil-moisture measurement is unavailable or invalid. The unusable value is omitted from the corresponding processed field, and the resulting database entry is assigned the PARTIAL processing status.

**Figure 7:**
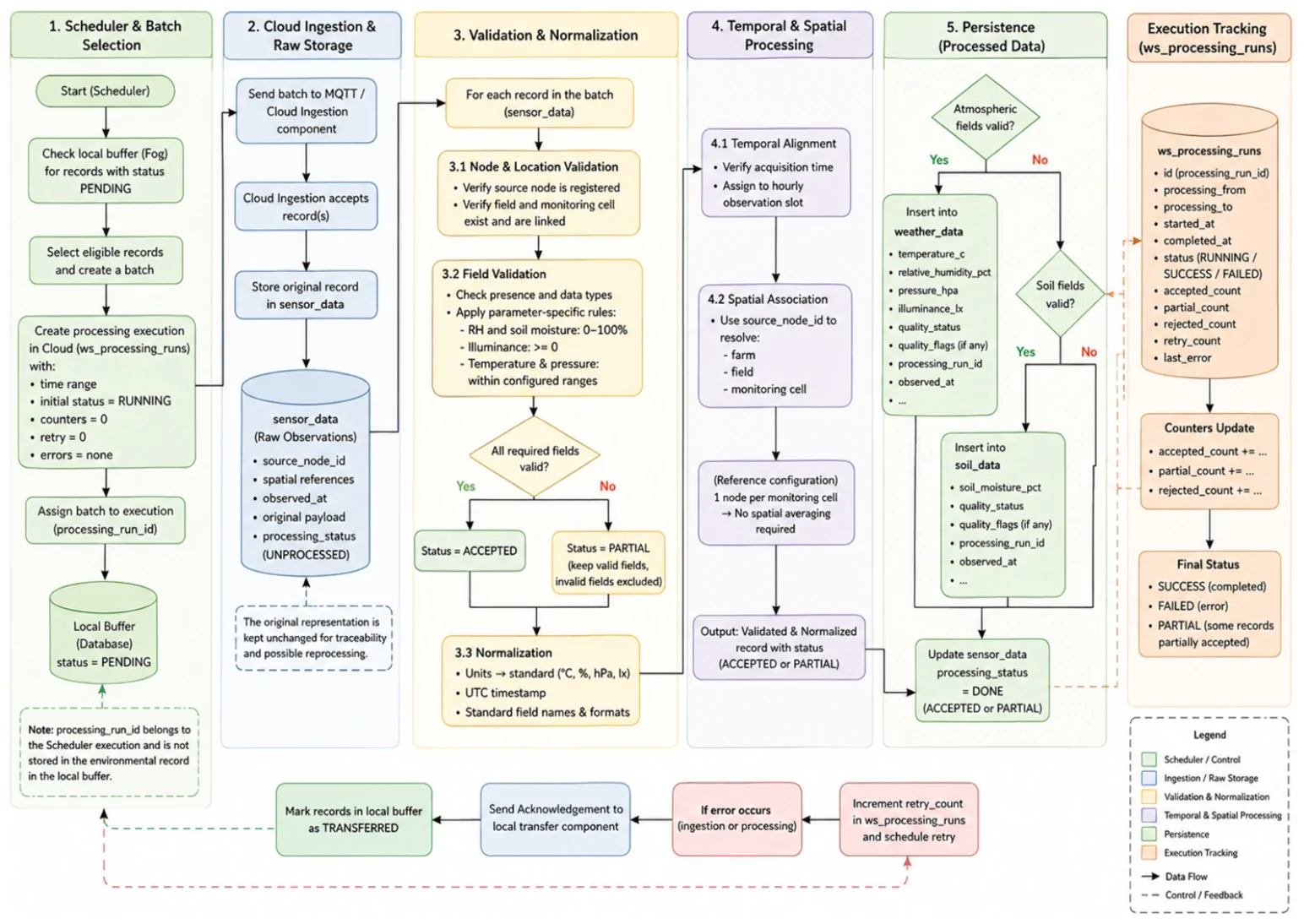
Scheduler-controlled Weather & Soil processing workflow, from Fog-level batch selection and Cloud ingestion to validation, normalization, spatial association, database persistence, and execution tracking.

Figure 7 summarizes the complete Scheduler-controlled processing workflow. Although the preceding paragraphs describe the batch-selection and validation stages in detail, the figure also shows the subsequent normalization, temporal and spatial processing, database persistence, execution tracking, acknowledgement, and retry operations.

As shown in Figure 7, validation represents one stage of a broader processing sequence. Records that contain at least one usable environmental value continue to the normalization stage, whereas records that cannot be processed are retained with the corresponding execution outcome. The following paragraphs describe the normalization and temporal–spatial processing stages represented in the central part of the workflow.

Normalization converts accepted measurements into the standard representation used by the FMIS. The system records air temperature in degrees Celsius; relative humidity and soil moisture as percentages; atmospheric pressure in hectopascals; and illuminance in lux. All acquisition times use a shared UTC-based timestamp. It also standardizes field names and numeric representations, regardless of the format produced by the local acquisition software [24,25,26].

The second processing stage deals with organizing validated observations in time and space. Since each node produces one environmental record per hour, the current implementation does not derive hourly averages, minimums, or maximums from multiple samples. Instead, temporal processing only verifies the acquisition time and places each record in its corresponding hourly observation slot.

Spatial processing starts with the source-node identifier, using it to locate the node registration and establish the associated farm, field, and monitoring cell. In the reference configuration, each monitoring cell has one acquisition node, so there is no need to average data spatially across multiple nodes. The node’s reported values are therefore recorded as the environmental conditions observed in that cell. Future configurations could use shorter sampling intervals or multiple nodes per cell; such changes would allow temporal or spatial statistical aggregation to be introduced without altering the Fog-to-Cloud transfer format.

In the present implementation, the temporal and spatial aggregation stage reduces to hourly alignment and monitoring-cell association because one record is generated per node and hour and one node is assigned to each monitoring cell.

**Figure 8:**
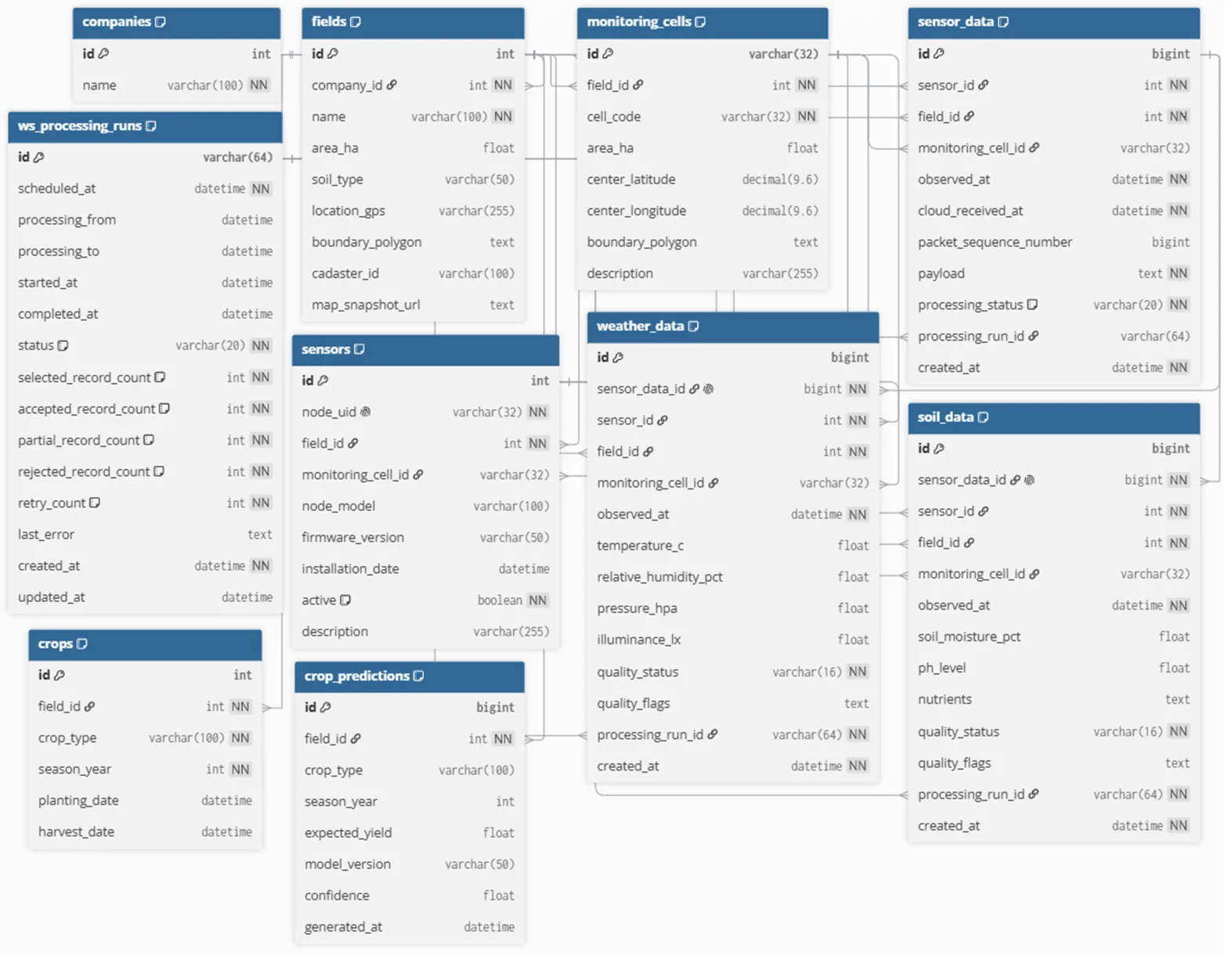
Relational Cloud database schema supporting the Weather & Soil processing workflow.

The persistence stage separates the original Cloud input from the validated domain data. Figure 8 presents the relational database segment used by the Weather & Soil workflow. The schema includes the raw observation table, the processed weather and soil tables, the node and spatial-registration entities, and the execution table used to track Scheduler-controlled processing runs.

As shown in Figure 8, each Cloud observation is first stored in sensor_data. The relation with the sensors table identifies the acquisition node, while field_id and monitoring_cell_id preserve the spatial context received from the local infrastructure or resolved from the node registration. The original payload remains available in sensor_data, together with the processing status and the Scheduler execution that handled it.

After validation and normalization, atmospheric measurements are written to weather_data. For the sensing configuration used in this study, this table contains air temperature, relative humidity, atmospheric pressure, and ambient illuminance. The corresponding soil-moisture value is written to soil_data. Both processed tables reference the same sensor_data row, which makes it possible to trace the stored environmental values back to the original buffered record.

The ws_processing_runs table stores the operational state of each Scheduler-controlled execution. Its fields record the selected time range, start and completion times, current status, number of accepted, partially accepted, and rejected records, retry count, and the last processing error. These data allow incomplete executions to be identified and repeated without losing the original observations.

The crop_predictions table is shown to illustrate the downstream use of the processed environmental data, but it is not written by the Weather & Soil service. Instead, the Prediction service reads the validated entries from weather_data and soil_data and stores its results independently.

The Scheduler controls the provided data for execution of the Weather & Soil workflow runs, but it does not perform the environmental calculations. On each configured run, it finds the records eligible for processing, creates a ws_processing_runs entry, and submits the relevant batch to the service. Processing takes place in the Weather & Soil service, which reports how many observations were accepted, partially accepted, or rejected. Those counters, along with the execution status, are then recorded in ws_processing_runs.

Records are handled idempotently: combining the unique record_id with the source-node identifier and acquisition timestamp ensures that retransmitting a batch does not create duplicate entries for the same observation. When a transfer or processing step fails, the original record stays either in the local buffer or in sensor_data, depending on where the failure happened. The Scheduler can then retry the operation, increment the retry counter, and retain the diagnostic information from the failed run.

Once processing succeeds, the Cloud confirms that the corresponding records are complete. The local transfer component marks those records as TRANSFERRED. If no acknowledgement arrives, they remain pending or failed and are included in a later retry. As a result, field acquisition remains separate from Cloud availability, so temporary communication or service interruptions do not remove measurements that have not yet been processed.

In addition to environmental records, the Weather & Soil service receives device-health and radio-communication telemetry generated at separate reporting intervals. These records contain battery, power, uptime, RSSI, SNR, packet-counter, relay, and channel-utilization information. They follow the same Scheduler-controlled transfer and validation mechanism but are persisted in dedicated monitoring tables.

## 6. Experimental Results

This chapter presents the experimental evaluation of the proposed LoRa Mesh environmental monitoring platform. The objective of the experimental campaign was twofold. First, the complete sensing platform was deployed under real outdoor conditions in order to validate the operation of the embedded hardware, LoRa Mesh communication subsystem, and gateway infrastructure. Second, representative environmental datasets were processed through the proposed acquisition and communication pipeline to evaluate the end-to-end functionality of the system, including telemetry transmission, data forwarding, and cloud integration.

The experimental evaluation therefore combines real communication measurements collected from the deployed hardware with representative environmental observations used to demonstrate the complete data-processing workflow. The evaluation focuses on the tested sensing-node-to-router configuration and on the end-to-end integration of the acquisition, communication, and cloud-processing components.

### 6.1. Experimental Deployment

The experimental platform was assembled using two Heltec Mesh Node T114 development boards configured with different roles within the proposed architecture. The first board was configured as an environmental sensing node and interfaced with the BME280, TEMT6000, and capacitive soil moisture sensors, while the second board was configured as a dedicated LoRa Mesh router operating simultaneously as the network gateway. The sensing node periodically acquired environmental measurements and transmitted telemetry packets through the Meshtastic communication framework, whereas the router continuously received the incoming packets and forwarded the recovered telemetry to the local server via the USB CDC interface.

**Figure 9:**
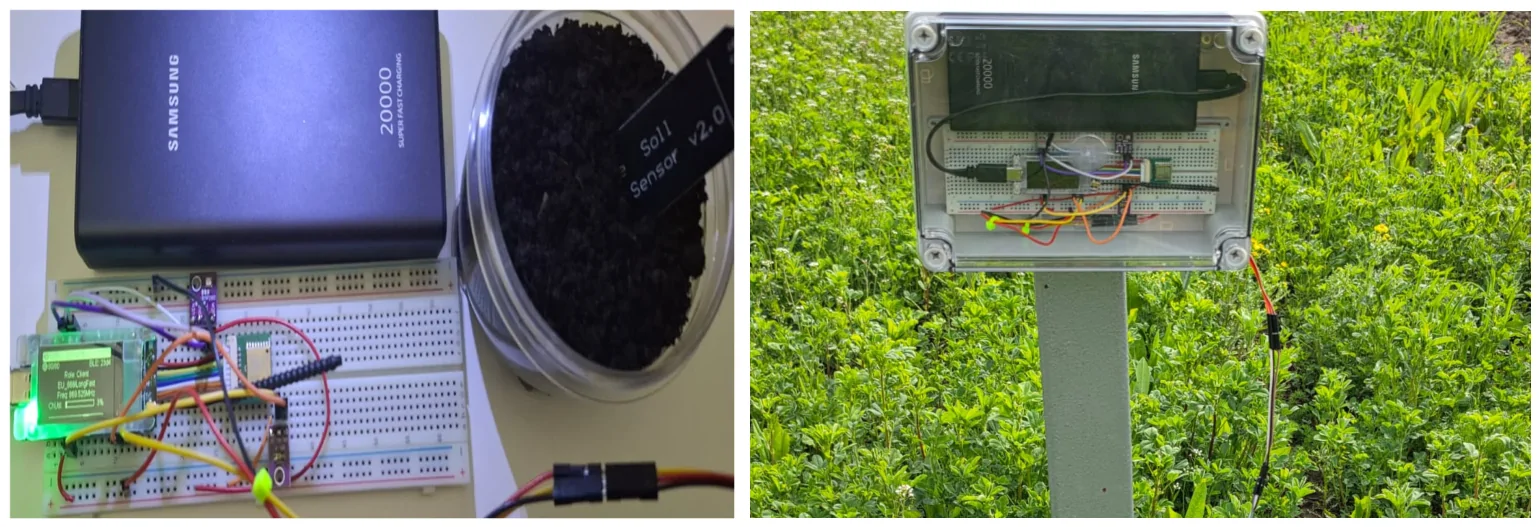
Experimental implementation of the proposed environmental monitoring node. (**left**) Laboratory prototype assembled on a solderless breadboard for hardware integration and firmware validation. (**right**) Outdoor deployment of the sensing node inside a weather-resistant enclosure during field testing.

Figure 9 illustrates the implementation stages of the proposed sensing platform. Initially, the complete hardware prototype was assembled on a solderless breadboard to validate the sensor interfaces, embedded firmware, and LoRa Mesh communication subsystem under laboratory conditions (Figure 9-left). After successful functional verification, the sensing node was integrated into a weather-resistant enclosure and deployed in an outdoor agricultural environment, as shown in Figure 9-right. The capacitive soil moisture sensor was inserted directly into the soil, while the enclosure protected the embedded electronics and power supply during field operation.

This two-board configuration should be interpreted as a proof-of-concept evaluation of the sensing-to-gateway communication path and the end-to-end platform workflow. It was not intended to demonstrate multi-hop mesh performance or network scalability, which require larger deployments involving multiple sensing and router nodes.

The experimental deployment was carried out under outdoor conditions in an agricultural environment in order to validate the complete sensing, communication, and gateway infrastructure. Throughout the experimental campaign, the router node remained continuously active, while the sensing node periodically performed environmental acquisition and wireless telemetry transmission according to the firmware workflow presented in Section 4. The gateway continuously collected the received telemetry packets and forwarded them to the local server, where the measurements were stored in a PostgreSQL database for subsequent analysis.

To complement the communication experiment, a 30-day environmental dataset was collected using the proposed sensing platform in the Bucharest region. The dataset contains measurements acquired by the integrated BME280, TEMT6000, and capacitive soil-moisture sensors. The collected data were used to evaluate the longer-term behavior of the environmental data-acquisition and processing workflow and to analyze the temporal evolution of the monitored environmental parameters under realistic operating conditions.

### 6.2. Environmental Measurements

**Figure 10:**
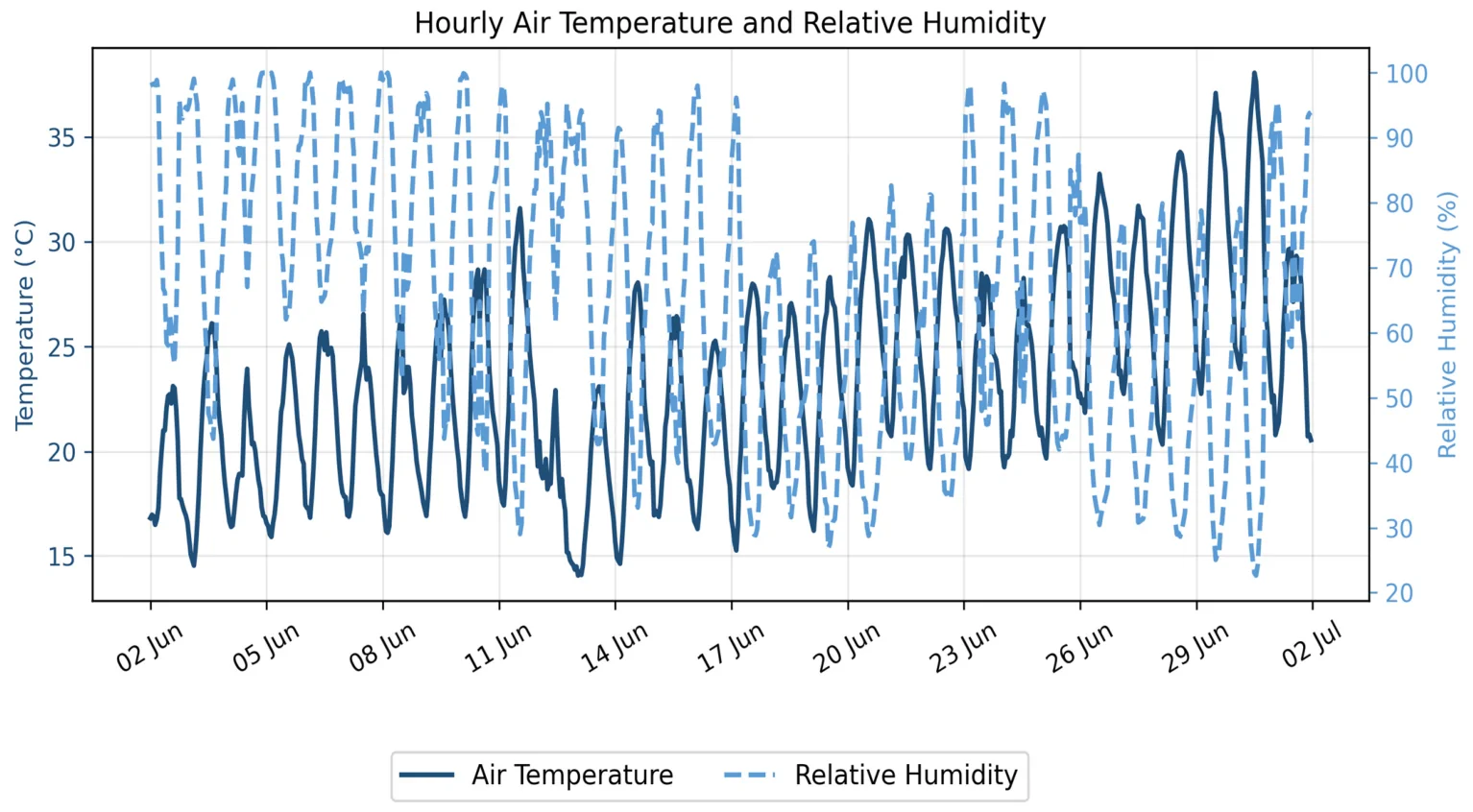
Air temperature and relative humidity.

**Figure 11:**
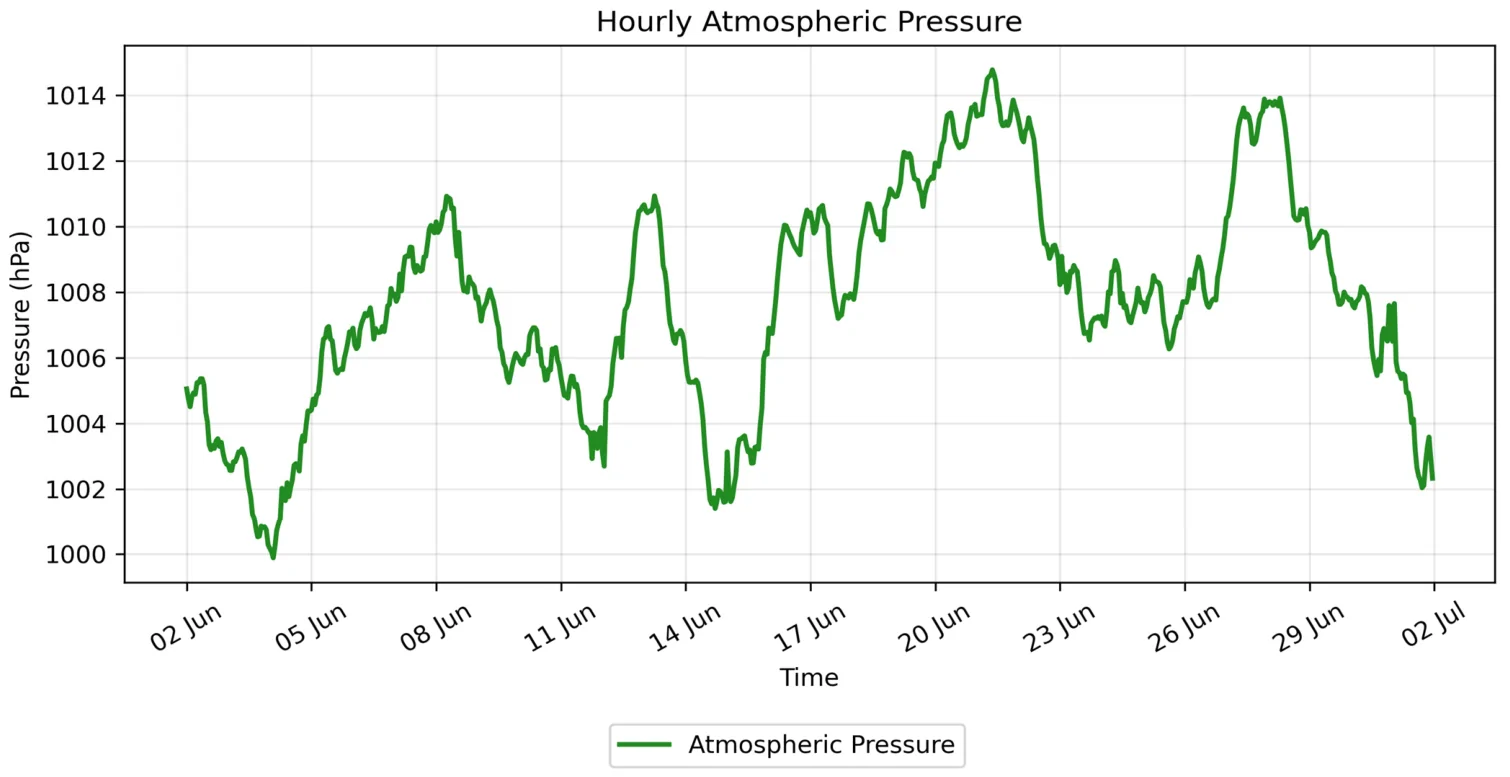
Atmospheric Pressure.

**Figure 12:**
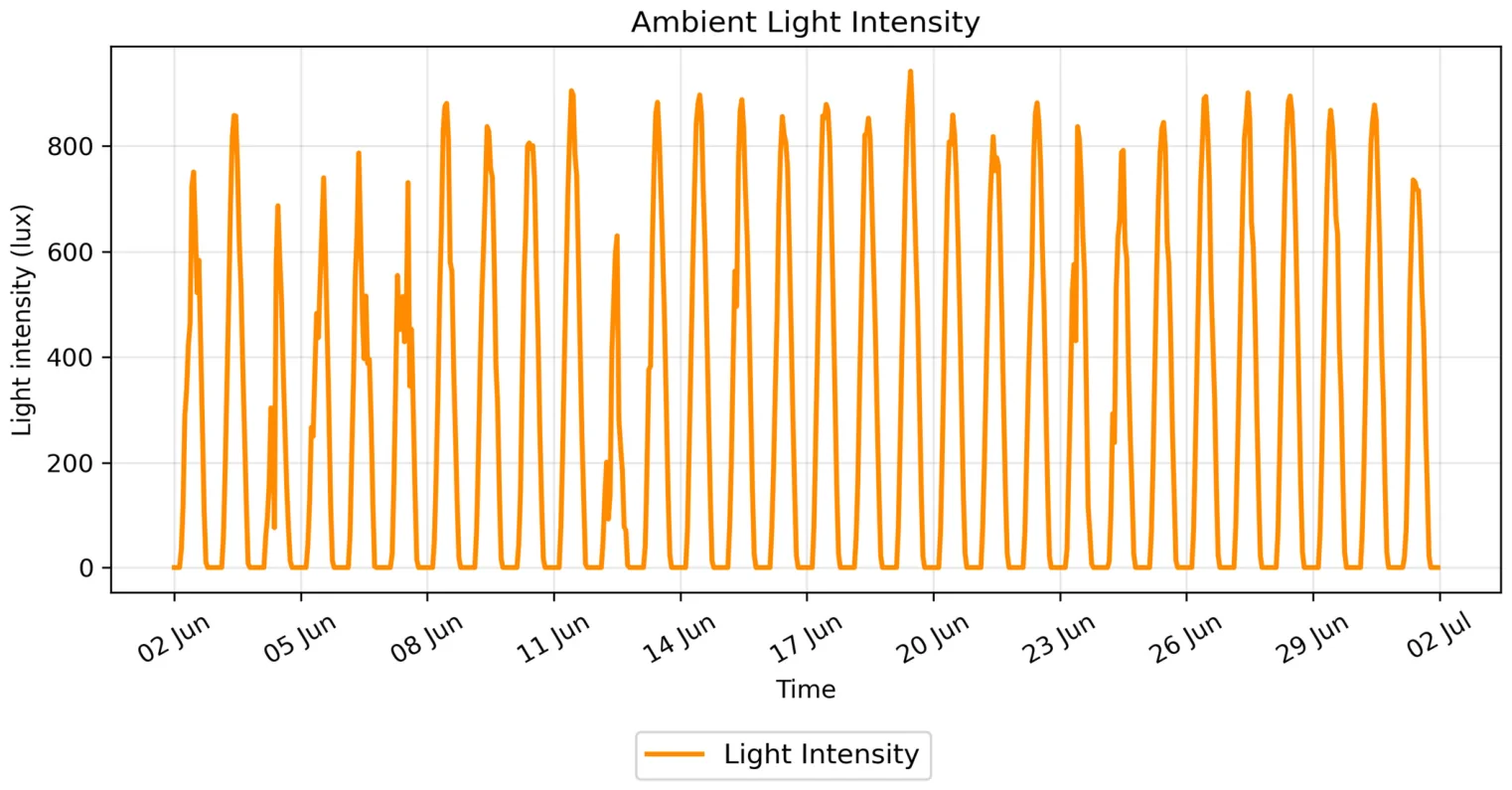
Ambient Light Intensity.

**Figure 13:**
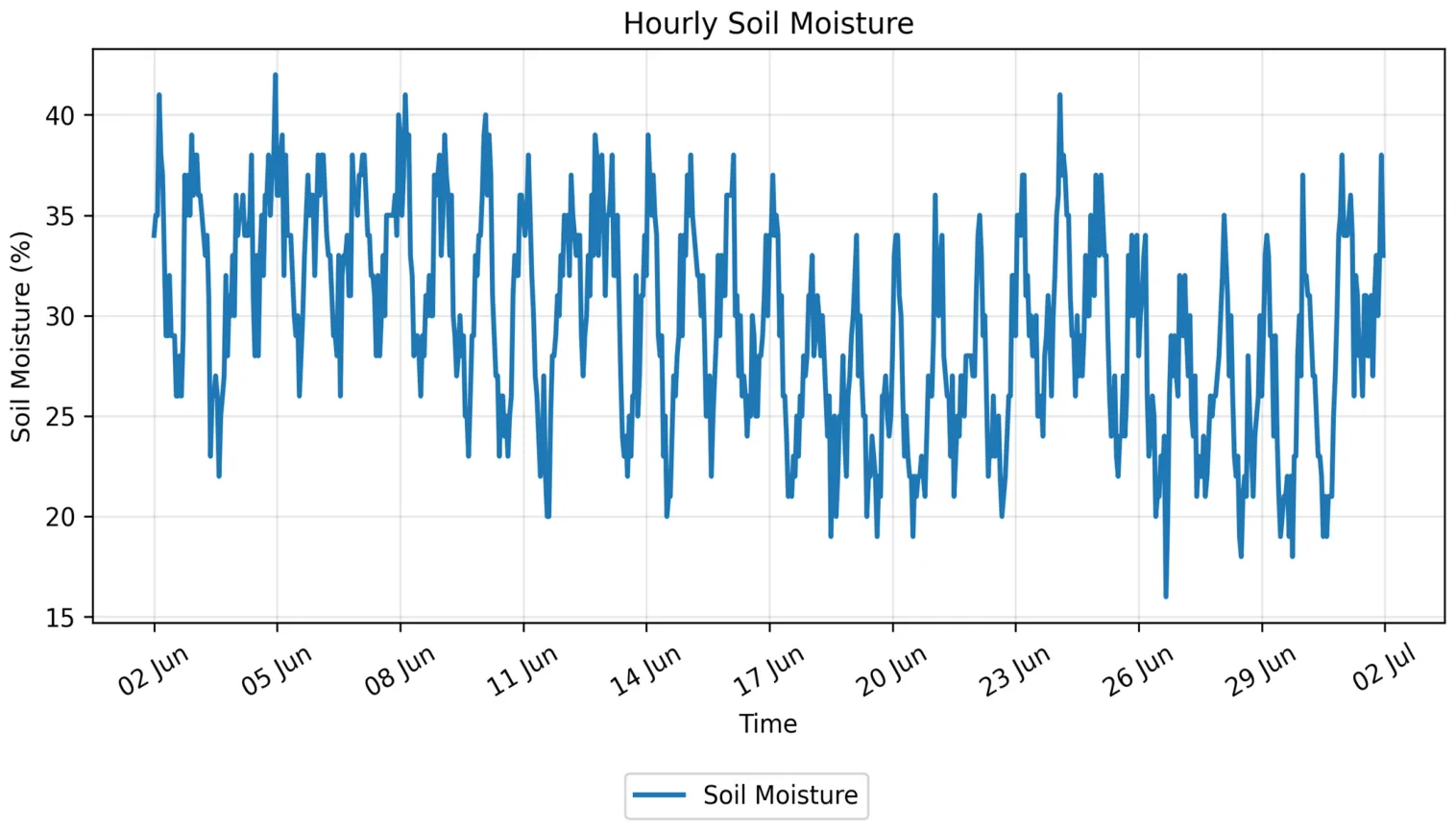
Soil Moisture.

The environmental measurements collected by the proposed monitoring platform are analyzed in this section to evaluate the sensing performance of the embedded acquisition system. Figure 10, Figure 11, Figure 12 and Figure 13 present the temporal evolution of the monitored environmental parameters throughout the evaluation period, including air temperature, relative humidity, atmospheric pressure, ambient light intensity, and soil moisture. Together, these measurements provide a comprehensive representation of the environmental conditions while demonstrating the capability of the proposed platform to continuously acquire, store, and process heterogeneous environmental data. The observed temporal variations are consistent with expected meteorological behavior and support the continuous operation of the sensing hardware, firmware, and data-acquisition pipeline under the tested conditions.

Figure 10 illustrates the hourly variation in air temperature and relative humidity during the 30-day evaluation period. As expected, the two parameters exhibit a clear inverse relationship throughout the daily cycle. Following sunrise, increasing solar radiation gradually raises the air temperature, while the relative humidity decreases because warmer air is capable of retaining a larger amount of water vapor. During the evening and nighttime hours, the cooling of the atmosphere is accompanied by a gradual increase in relative humidity, with the highest values occurring during the early morning. The measured trends are consistent with typical meteorological conditions observed in temperate climates and show that the proposed sensing platform tracks short-term variations in air temperature and relative humidity over the observation period.

The mean air temperature was approximately 23.4 °C (SD = 4.8 °C), with values ranging from about 14.0 to 38.1 °C, while relative humidity averaged approximately 66.2% (SD = 20.7%), with values ranging from about 22% to 100%. The two parameters exhibit a clear inverse relationship throughout the daily cycle. Following sunrise, increasing solar radiation gradually raises the air temperature, while relative humidity decreases. During the evening and nighttime hours, cooling is accompanied by a gradual increase in relative humidity. The observed trends show that the sensing platform tracks short-term variations in air temperature and relative humidity over the observation period.

Figure 11 presents the atmospheric pressure measurements acquired during the experimental evaluation. Unlike temperature and humidity, atmospheric pressure changes gradually over time and remains within a relatively narrow operating interval. The recorded pressure profile exhibits only minor fluctuations, indicating stable weather conditions throughout most of the observation period. Such behavior confirms both the stability of the BME280 sensing module and the robustness of the proposed acquisition pipeline during continuous long-term monitoring.

The mean atmospheric pressure was approximately 1007.8 hPa (SD = 3.3 hPa), with values ranging from about 999.9 to 1014.8 hPa. Compared with temperature and humidity, atmospheric pressure varied more gradually and remained within a relatively narrow interval throughout the observation period.

Figure 12 shows the evolution of ambient light intensity measured by the TEMT6000 sensor. The recorded measurements clearly reproduce the expected day–night cycle, with illumination levels approaching zero during nighttime and reaching maximum values around midday. Daily variations in peak light intensity can also be observed and are mainly associated with changing atmospheric conditions, including cloud cover and solar irradiance. The obtained measurements show that the optical sensing subsystem tracks diurnal illumination patterns relevant to agricultural monitoring.

The mean recorded illuminance was approximately 286 lx (SD = 295 lx), with values ranging from approximately 0 to 944 lx. The temporal profile reflects a pronounced day–night cycle, with near-zero values during nighttime and maximum values occurring during daytime periods.

Figure 13 presents the normalized soil moisture measurements obtained using the capacitive soil moisture sensor. Compared with the atmospheric parameters, soil moisture evolves considerably more slowly due to the higher water retention capacity of the soil. The relatively smooth temporal profile indicates gradual changes in water availability rather than rapid fluctuations, reflecting the physical characteristics of soil moisture dynamics. Such measurements are particularly valuable for irrigation management, where long-term trends are generally more relevant than short-term variations.

The mean soil-moisture level was approximately 29.6% (SD = 4.7%), with values ranging from about 16.2% to 42.0%. Compared with the atmospheric parameters, soil moisture exhibited slower temporal variations, with gradual changes in water availability observed throughout the monitoring period.

Overall, the analyzed environmental parameters exhibit temporal variations consistent with the expected behavior of outdoor agricultural environments. The absence of anomalous discontinuities and the coherence between correlated variables, such as air temperature and relative humidity, indicate reliable operation of the sensing hardware and embedded firmware throughout the evaluation period. These results confirm that the proposed platform is capable of performing continuous environmental monitoring while providing stable and consistent measurements suitable for precision agriculture applications.

### 6.3. Communication Performance

The communication performance of the proposed Meshtastic-based LoRa platform was experimentally evaluated during a continuous four-hour outdoor deployment. Device telemetry and local communication statistics were collected at one-minute intervals using the telemetry services provided by the Meshtastic framework. This acquisition interval provided approximately 240 measurement points over the four-hour experiment, allowing the temporal evolution of the radio-link and device-level parameters to be observed at relatively fine resolution. Unlike conventional network performance evaluations based exclusively on packet reception statistics, the proposed approach combines radio-quality indicators, channel occupancy metrics, device operating parameters, and local communication statistics. Consequently, the communication subsystem can be evaluated from multiple complementary indicators without introducing additional diagnostic traffic or modifying normal network operation. The four-hour experiment should nevertheless be interpreted as an initial short-term communication stability evaluation rather than as evidence of long-term communication reliability.

**Figure 14:**
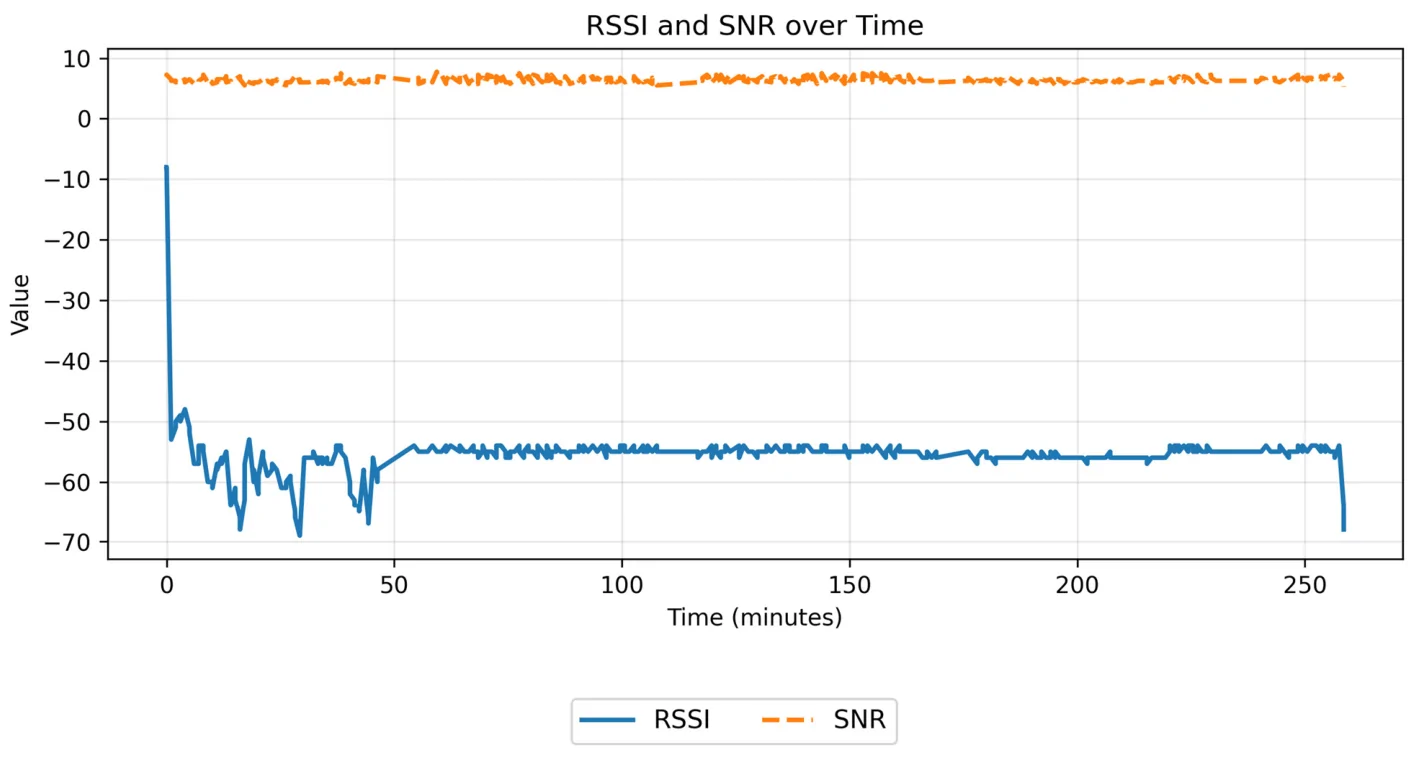
Temporal evolution of the received signal strength indicator (RSSI) and signal-to-noise ratio (SNR).

Figure 14 presents the temporal evolution of the received signal strength indicator (RSSI) and signal-to-noise ratio (SNR) measured during the communication experiment. The measurements shown in Figure 14 correspond to the short-range outdoor test performed in an open-field environment, with no significant obstacles between the sensing node and the router/gateway. Both devices operated in the 868 MHz band and used the standard external LoRa antennas supplied with the Heltec Mesh Node T114 boards. After a short initialization interval, both radio parameters remained within relatively stable operating ranges.

The RSSI stabilized around −55 dBm, with typical short-range values between approximately −55 and −60 dBm, while the SNR remained close to 6–7 dB throughout the evaluation period. An additional four-hour communication observation was performed over a distance of approximately 4 km, where the propagation path included both open-field sections and a built-up residential area containing houses and other obstacles. During this long-distance test, the average RSSI was approximately −85.7 dBm (SD = 11.4 dB), with values ranging from −101 to −73 dBm, while the average SNR was approximately 5.43 dB (SD = 0.67 dB), with values between 4.0 and 6.0 dB. Channel utilization averaged approximately 11.29%, ranging from 9.88% to 15.49%, while transmission airtime utilization averaged approximately 1.42%, with values between 1.02% and 1.98%. This additional test was focused on characterizing long-distance radio-link quality and channel usage under mixed open-field and built-up propagation conditions. Minor short-term fluctuations observed during the short-range experiment are attributed to normal variations in the wireless propagation channel and environmental conditions. The isolated RSSI transient observed during system startup corresponds to the initialization phase of the communication subsystem. Overall, the measurements indicate stable short-term radio-link behavior under the tested propagation conditions.

**Figure 15:**
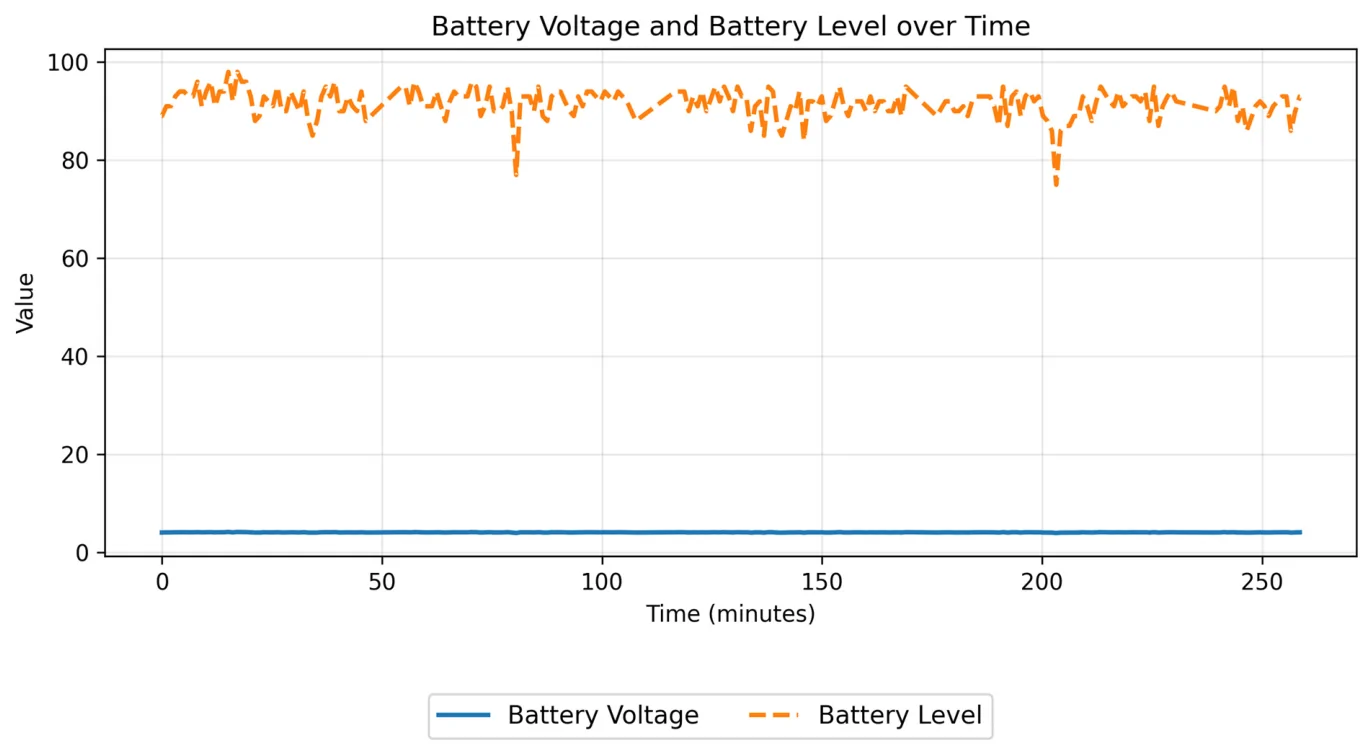
Temporal evolution of the battery voltage and estimated battery charge level during continuous platform operation.

Figure 15 illustrates the evolution of the battery voltage together with the estimated battery charge level reported by the embedded telemetry service during the four-hour communication experiment. The measured battery voltage remained nearly constant, varying only within a narrow interval around 4.1 V, indicating stable power supply conditions throughout continuous operation. Small fluctuations observed in the reported battery percentage are expected because the battery level is internally estimated from the measured voltage and therefore reflects minor voltage variations caused by the transmission activity and the battery management circuitry. No pronounced downward trend in battery voltage was observed during the four-hour experiment, indicating stable supply conditions over the evaluated interval. This behavior is consistent with the low-duty-cycle operating strategy of the sensing node; however, battery-voltage stability alone does not provide a quantitative measure of energy consumption or long-term battery autonomy.

It is worth noting that the communication experiment was intentionally configured with one-minute telemetry intervals to facilitate continuous performance evaluation. Under the intended operating conditions, where environmental measurements are acquired once per hour, the sensing node remains in low-power sleep mode for most of the time. Therefore, the actual energy consumption is expected to be considerably lower, enabling substantially longer battery lifetime.

**Figure 16:**
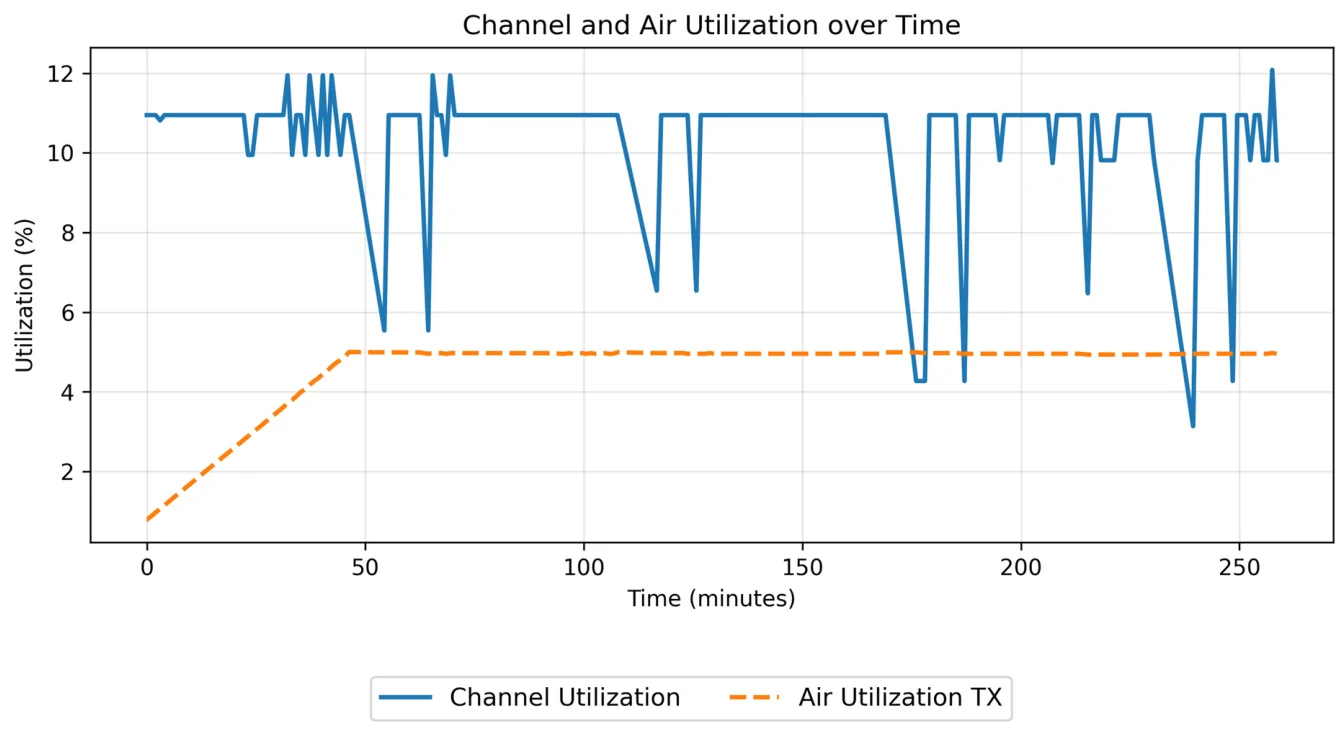
Evolution of channel utilization and transmission airtime utilization reported by the Meshtastic communication framework.

Figure 16 presents the temporal evolution of the channel utilization and transmission airtime utilization reported by the Meshtastic communication stack. Throughout the experiment, the transmission airtime remained below 5%, indicating that only a small fraction of the available radio airtime was occupied by telemetry traffic. The channel utilization fluctuated between approximately 4% and 12%, reflecting normal variations in the measured channel occupancy and the adaptive estimation performed by the firmware. Temporary decreases do not indicate communication failures but rather correspond to variations in the estimated activity of the wireless medium. Overall, the observed utilization levels indicate that the tested single-node telemetry configuration generated only a modest communication load. These measurements suggest that additional channel capacity may be available; however, the number of simultaneously active sensing nodes that can be supported was not experimentally evaluated in the present study.

**Figure 17:**
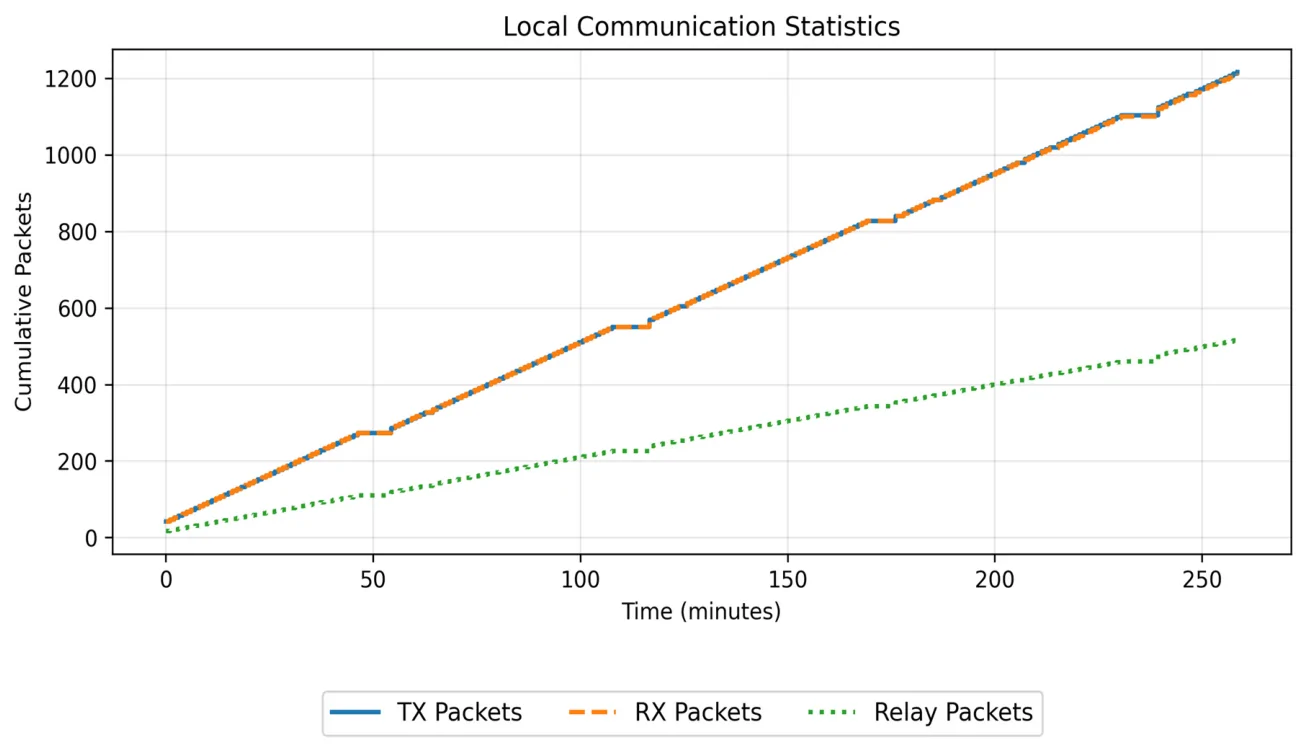
Evolution of cumulative transmitted packets, received packets, and relay packets during the communication experiment.

Figure 17 summarizes the cumulative communication statistics collected by the Meshtastic firmware during the experiment. Both transmitted and received packet counters increased almost linearly, confirming the periodic generation and successful reception of telemetry packets throughout the entire measurement interval.

During the approximately four-hour evaluation interval, the cumulative communication counters reached 1124 transmitted packets and 1120 received packets. This corresponds to an RX/TX ratio of approximately 99.6%. In addition, the firmware reported no bad received packets and no dropped transmitted packets during the evaluated interval. These values provide a quantitative indication of the stable packet exchange observed in the tested sensing-node-to-router configuration.

The relay packet counter increased more slowly, as expected, because the experimental deployment consisted of a single sensing node communicating directly with one router node, requiring only a limited number of relay operations for network management. These results confirm the correct operation of the packet scheduling mechanism, the routing services, and the overall communication subsystem under continuous operation.

Overall, the communication experiment showed stable operation of the tested sensing-node-to-router configuration. The measured RSSI and SNR values remained within consistent ranges, while the observed channel and transmission-airtime utilization indicated a modest communication load under the evaluated conditions. The TX and RX counters also showed consistent packet exchange throughout the experiment. Together, these results support the practical suitability of the proposed communication architecture for environmental monitoring and provide a useful basis for extending the platform toward longer-term and larger multi-node deployments.

## 7. Conclusions

This paper introduced a low-cost agricultural monitoring platform built around embedded environmental sensors, Meshtastic-based LoRa communication, and an Edge–Fog–Cloud software architecture for precision-agriculture monitoring applications. Commercially available hardware, open-source firmware, Fog-level preprocessing, and cloud microservices are brought together in one end-to-end workflow, covering environmental telemetry acquisition, transmission, buffering, processing, storage, and visualization. Rather than relying on proprietary infrastructure or centralized LoRaWAN deployments, the platform extends the Meshtastic framework for environmental sensing while retaining compatibility with its native mesh-networking mechanisms.

The central scientific and technical contribution of this work lies in designing and experimentally evaluating an interoperable architecture that combines heterogeneous environmental sensing with Meshtastic-based LoRa Mesh communication, queue-based transmission scheduling, local data buffering, and cloud-level processing. Meshtastic is often used for messaging and position reporting, but the implementation described here extends its telemetry framework to collect and carry environmental measurements. By separating sensing from radio transmission through the internal txQueue mechanism, the system can continue acquiring data even when the channel is unavailable. The architecture divides processing among its components: field nodes handle periodic sequential acquisition and signal conversion; the Fog layer carries out preliminary validation and persistent buffering; and the Weather & Soil microservice manages domain-level validation, normalization, spatial association, and cloud persistence.

A comparison with commercial smart-agriculture ecosystems further clarifies the positioning of the proposed platform. Solutions such as Milesight Smart Agriculture, Seeed Studio SenseCAP, and Libelium Smart Agriculture provide mature combinations of environmental sensing, LoRaWAN connectivity, gateway infrastructure, device management, and cloud-based monitoring. Milesight offers dedicated agricultural sensing devices, LoRaWAN gateways, and cloud-based management services, while SenseCAP combines sensing devices, gateways, cloud services, and integration interfaces; Libelium similarly provides smart-agriculture solutions covering atmospheric, soil, irrigation, and weather-related monitoring. In comparison, the proposed platform follows a more research-oriented and customizable approach in which sensing hardware, Fog-level data handling, and cloud-side processing can be adapted independently. The use of commercially available embedded components and open-source software also provides the potential for a lower initial deployment cost and incremental expansion according to monitoring requirements. Commercial platforms, however, retain important advantages in terms of turnkey deployment, industrial-grade hardware, certification, and mature device-management services.

From an economic standpoint, the platform is built around commercially available embedded boards, inexpensive environmental sensors, open-source firmware, and a shared LoRa Mesh infrastructure. Based on the prototype configuration used in this study, the indicative hardware cost of one sensing node is approximately EUR 45–65, depending on supplier and component configuration. This estimate includes the embedded board, environmental sensors, battery, and basic interconnection components, while enclosure, installation, router/gateway infrastructure, and server-side equipment are excluded. The compatibility with Meshtastic multi-hop mechanisms provides an architectural basis for future deployments in which additional router nodes may extend communication coverage without requiring a dedicated gateway at every monitoring location. Duty-cycled acquisition and low-power radio communication also keep energy use and maintenance demands down. The modular architecture makes gradual deployment possible: sensing and router nodes can be added where greater spatial resolution or wider communication coverage is needed, rather than installed everywhere from the outset.

The experiments showed that the proposed architecture operated consistently under the tested conditions. Its sensing subsystem tracked temporal variations in air temperature, relative humidity, atmospheric pressure, soil moisture, and ambient illuminance, while the communication subsystem maintained stable RSSI and SNR values, low channel occupancy, and consistent packet transmission throughout the monitoring period. Battery voltage also remained stable during the evaluated interval. Together with the duty-cycled operating strategy, these observations support the practical feasibility of battery-powered sensing nodes for agricultural monitoring scenarios where permanent access to the electrical grid may be limited. A quantitative characterization of long-term battery autonomy, however, requires dedicated current measurements and extended-duration experiments.

From an architectural perspective, the proposed platform has the potential to support larger monitoring areas without requiring fundamental changes to its core communication or processing components. New sensing nodes can join the existing LoRa Mesh network, and router nodes can be added in locations where terrain, vegetation, or distance makes direct communication with the gateway unreliable. Because Edge acquisition, Fog preprocessing, and cloud microservices are kept separate, software components can be revised or scaled individually rather than across the entire platform.

From an architectural perspective, the proposed platform has the potential to support larger monitoring areas through the addition of sensing and router nodes without requiring fundamental changes to the core sensing and data-processing workflow. New sensing nodes can be incorporated according to the required spatial resolution, while additional router nodes may be introduced where terrain, vegetation, or distance limits direct communication with the gateway. The separation of Edge acquisition, Fog preprocessing, and cloud microservices further supports this potential scalability, since individual software and infrastructure components can be extended independently according to deployment requirements. Larger multi-node and multi-hop deployments will be evaluated in future field experiments.

Future field-oriented validation will focus on longer-duration deployments under realistic agricultural conditions, considering factors such as terrain, vegetation, weather exposure, and node placement. These experiments will evaluate communication stability, channel contention, energy consumption, and sensing performance across multiple sensing and router nodes, while also addressing practical aspects related to weather-resistant installation and maintenance. Such evaluations will support the transition of the platform toward sustained use in real agricultural monitoring scenarios.

Further development of the cloud layer will center on environmental prediction, anomaly detection, irrigation assistance, and predictive maintenance for sensing nodes. Machine-learning models could draw on validated records from the Weather & Soil service, along with historical and spatial information. With these additions, the platform would extend beyond collecting telemetry and offer broader decision support for irrigation planning, resource management, and crop monitoring.

Overall, the study shows that open-source embedded hardware, Meshtastic-based LoRa Mesh communication, local store-and-forward processing, and cloud microservices can work together as a practical agricultural monitoring platform. Its architecture can support deployments from experimental plots through larger open-field installations, while allowing the system to expand gradually as sensing, communication, and decision-support needs change.

## Figures and Tables

**Table 1:** Comparison of wireless communication technologies used in agricultural IoT systems.

Parameter	Wi-Fi	Bluetooth	ZigBee	LoRa
Frequency	2.4 GHz	2.4 GHz	868/915 MHz, 2.4 GHz	433/868/915 MHz
Data Rate	High	Medium	Medium	Low
Communication Range	Short	Very Short	Medium	Long
Power Consumption	High	Medium	Low	Very Low
Network Topology	Star	Star	Mesh	Star/Mesh
Suitability for Agricultural Monitoring	Moderate	Limited	Moderate	High

Note: The communication characteristics presented in Table 1 are based on the comparative values reported in [1] and related agricultural IoT literature [5,6,7,8,9]. The terms “Very Low”, “Low”, “Medium”, and “High”, as well as the communication-range categories, are used as relative qualitative classifications for comparative purposes and do not represent standardized performance classes. Actual performance depends on the specific hardware implementation, radio configuration, transmission power, duty cycle, and deployment environment.

**Table 2:** Environmental sensors frequently used in agricultural monitoring systems.

Monitoring Category	Parameter	Example Sensors	Communication Interface
Environmental monitoring	Temperature and humidity	DHT22, BME280	I2C/Digital
Environmental monitoring	Atmospheric pressure	MPL3115A2, BME280	I2C
Soil monitoring	Soil moisture	Capacitive soil moisture sensor	ADC
Environmental monitoring	Luminosity	TEMT6000, BH1750	ADC/I2C
Environmental monitoring	CO_2_ concentration	MQ135, MG-811	Analog/ADC

**Table 3:** Electrical specifications of the embedded hardware components integrated in the proposed monitoring platform.

Component	Active Current	Sleep Current	Operating Voltage
nRF52840 MCU	~4.8 mA (CPU @ 64 MHz)	0.4–1.5 µA	1.7–5.5 V [22]
SX1262 LoRa Transceiver	4.6 mA (RX), up to 118 mA (+22 dBm TX)	160 nA	1.8–3.7 V [23]
BME280	~714 µA (measurement)	0.1 µA	1.71–3.6 V [24]
TEMT6000	<50 µA (typical)	N/A	2.7–5.5 V [25]
Capacitive Soil Moisture Sensor	5–10 mA (typical module)	N/A	3.3–5 V [26]

**Table 4:** Logical structure of the hourly environmental record stored in the Fog-level buffer.

Field	Type	Description
record_id	varchar (64)	Unique identifier of the buffered environmental record
record_type	varchar (32)	Record category; ENVIRONMENTAL for the record described here
source_node_id	varchar (32)	Identifier of the acquisition node, e.g., JSH6UGB4AZ
farm_id	integer	Identifier of the monitored farm
field_id	integer	Identifier of the agricultural field
monitoring_cell_id	varchar (32)	Monitoring cell assigned to the sensing node
observed_at	timestamp	Time at which the values were acquired
buffered_at	timestamp	Time at which the local server stored the record
payload.temperature_c	float	Air temperature in °C
payload.relative_humidity_pct	float	Relative humidity in %
payload.pressure_hpa	float	Atmospheric pressure in hPa
payload.illuminance_lx	float	Ambient illuminance in lx
payload.soil_moisture_pct	float	Normalized soil moisture in %
transfer_status	varchar (16)	PENDING, IN_TRANSFER, TRANSFERRED, or FAILED
retry_count	integer	Number of attempted Cloud transfers
packet_sequence_number	bigint	Sequence number used for duplicate detection

## Data Availability

The data presented in this study are available on request from the corresponding author.

## Abbreviations

The following abbreviations are used in this manuscript:

ADC	Analog-to-Digital Converter
CAD	Channel Activity Detection
CDC	Communications Device Class
CPU	Central Processing Unit
DIO	Digital Input/Output
FMIS	Farm Management Information System
FSK	Frequency Shift Keying
GPIO	General-Purpose Input/Output
I^2^C	Inter-Integrated Circuit
IoT	Internet of Things
JSON	JavaScript Object Notation
KB	Kilobyte
LPWAN	Low-Power Wide-Area Network
LoRa	Long Range
LoRaWAN	Long Range Wide Area Network
MB	Megabyte
MCU	Microcontroller Unit
MQTT	Message Queuing Telemetry Transport
PCB	Printed Circuit Board
PWM	Pulse Width Modulation
RAM	Random Access Memory
RSSI	Received Signal Strength Indicator
RTC	Real-Time Clock
RX	Receive
SAADC	Successive Approximation Analog-to-Digital Converter
SAR	Successive Approximation Register
SCL	Serial Clock Line
SDA	Serial Data Line
SNR	Signal-to-Noise Ratio
SoC	System-on-Chip
SPI	Serial Peripheral Interface
SX1262	Semtech SX1262 LoRa Transceiver
TEMT6000	Ambient Light Sensor
TWI	Two-Wire Interface
TX	Transmit
UART	Universal Asynchronous Receiver/Transmitter
USB	Universal Serial Bus
UTC	Coordinated Universal Time
Wi-Fi	Wireless Fidelity
XML	Extensible Markup Language
